# Supplementary material for: Benchmarking of a Bayesian single cell RNAseq differential gene expression test for dose–response study designs
Source: Nucleic Acids Res. 2022 Jan 21;50(8):e48. doi: 10.1093/nar/gkac019 (PMC9071439; doi:10.1093/nar/gkac019)
Supplement: gkac019_Supplemental_Files [file gkac019_supplemental_files.zip › scBT-Benchmarking-SupplementalFigs_121321.pdf]

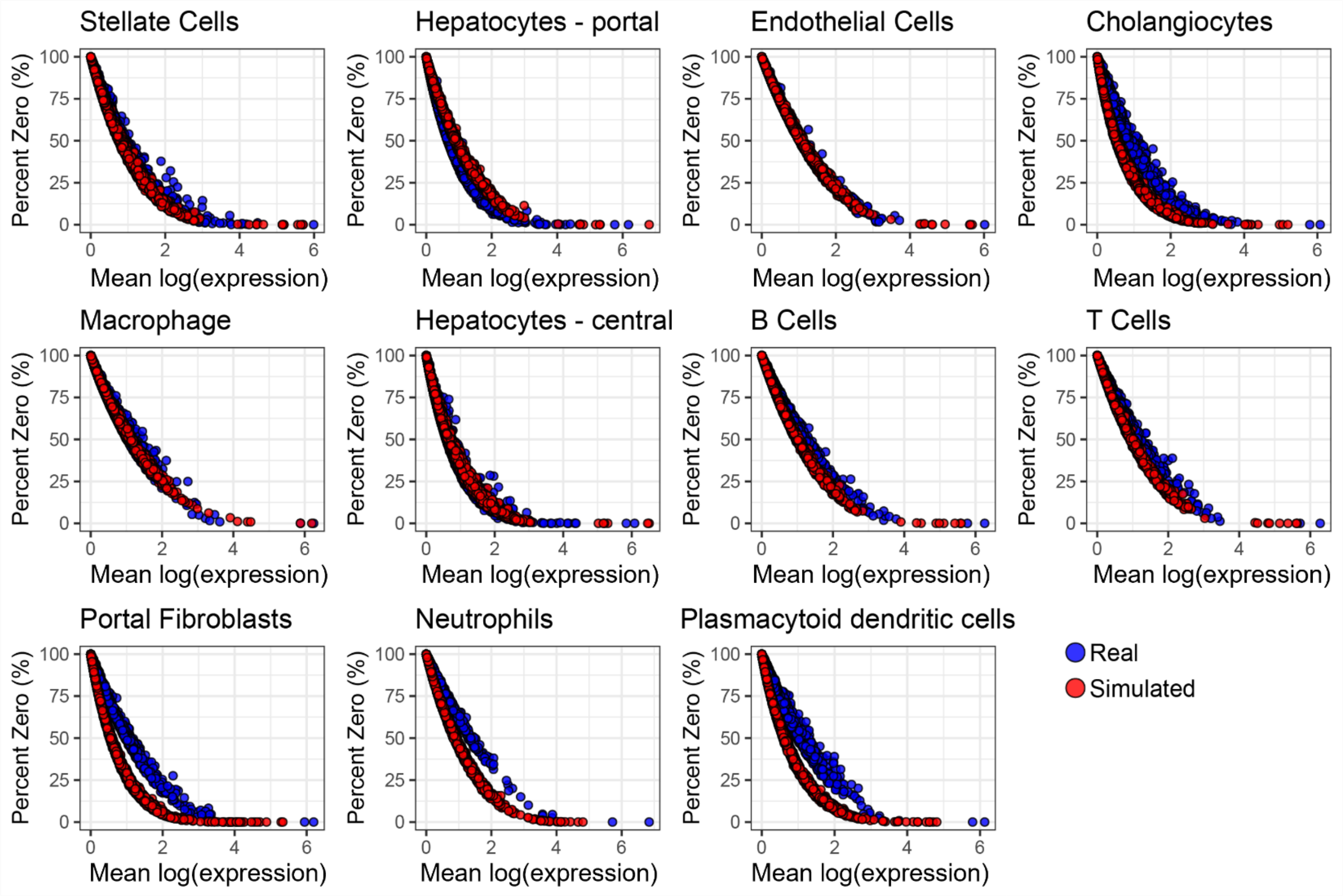

Supplementary Figure 1: Comparison of experimental and simulated dose-response data from cell specific initial parameters. Simulation parameters were estimated from each cell type identified in our experimental hepatic dose-response snRNAseq dataset. A total of 4500 cells (500 per dose) distributed across 3 individuals for 9 dose groups were simulated. The percent zeroes and mean log expression was calculated for each gene for all dose groups combined.

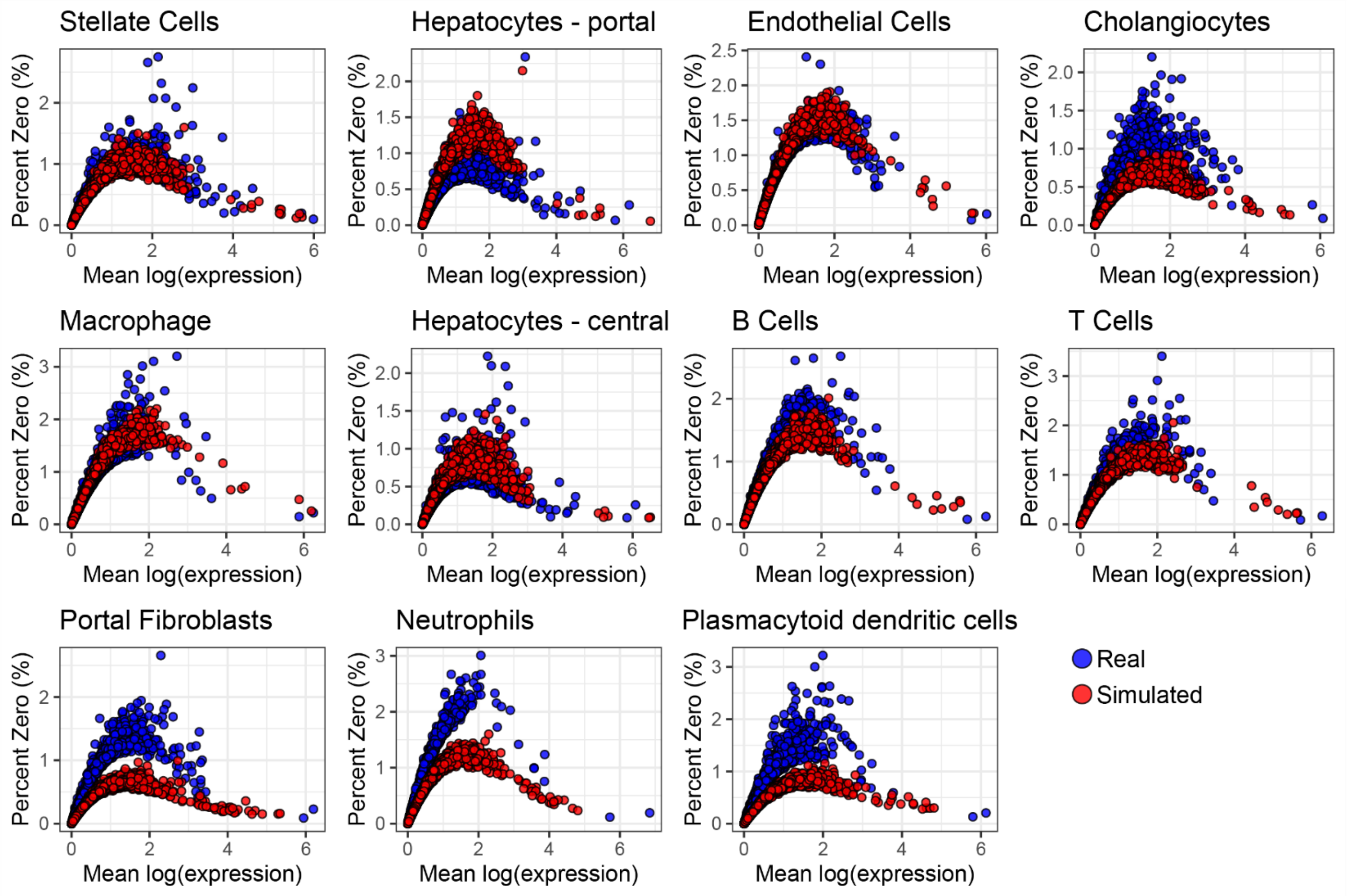

Supplementary Figure 2: Comparison of experimental and simulated dose-response data from cell specific initial parameters. Simulation parameters were estimated from each cell type identified in our experimental hepatic dose-response snRNAseq dataset. A total of 4500 cells (500 per dose) distributed across 3 individuals for 9 dose groups were simulated. The percent zeroes and mean log variance was calculated for each gene for all dose groups combined.

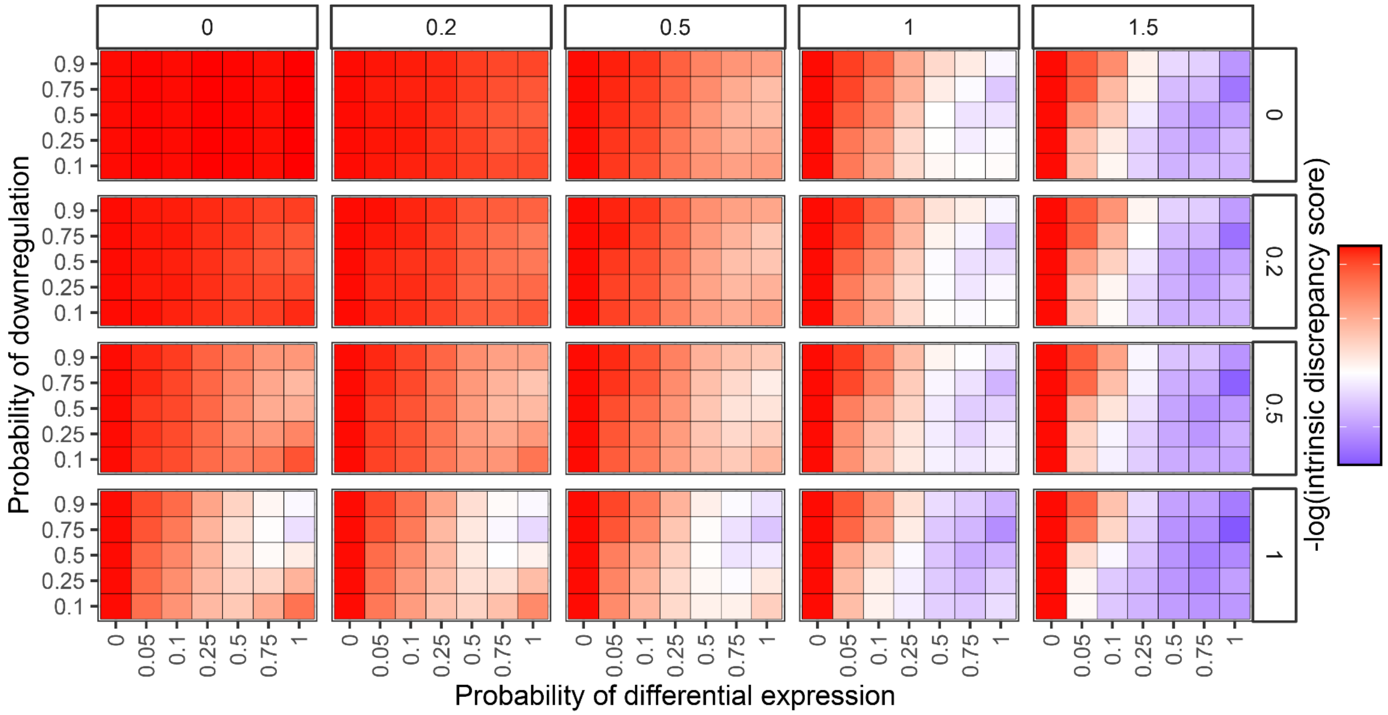

Supplementary Figure 3: Intrinsic discrepancy scores of fold-change distributions under varying simulation parameters. Datasets were simulated for 5,000 genes by varying the probability of differential expression, probability of repression, mean fold-change of differentially expressed genes (location) and distribution of fold-change for differentially expressed genes (scale). Fold-change location and scale of differentially expressed genes from 0 – 1.5 and 0 – 1, respectively, represent the values for mean and standard deviation of a log-normal distribution. 5,000 simulated genes were compared to an equivalent number sampled from experimental data for the fold-changes between 30 µg/kg TCDD and control groups. The Kullback-Leibler Divergence (KLD) intrinsic discrepancy (ID) was used to evaluate the similarity in distributions.

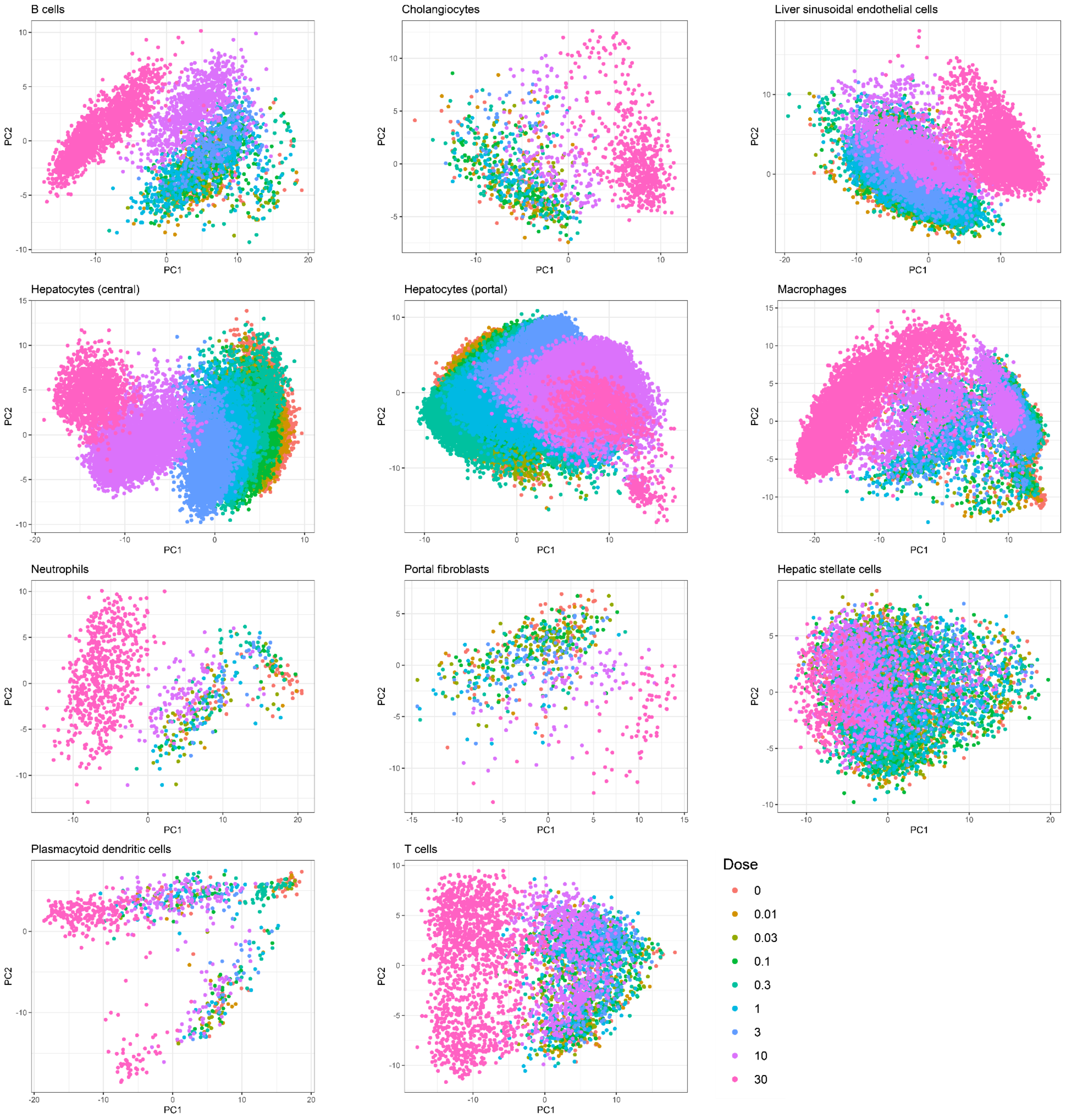

Supplementary Figure 4: Principal components analysis (PCA) of experimental hepatic dose-response data from male mice gavaged with 2,3,7,8-tetrachlorodibenzo-p-dioxin (TCDD) every 4 days for 28 days. PCA was performed for all genes and each identified cell type. Each point represents an individual cell. Colors represent treatment groups.

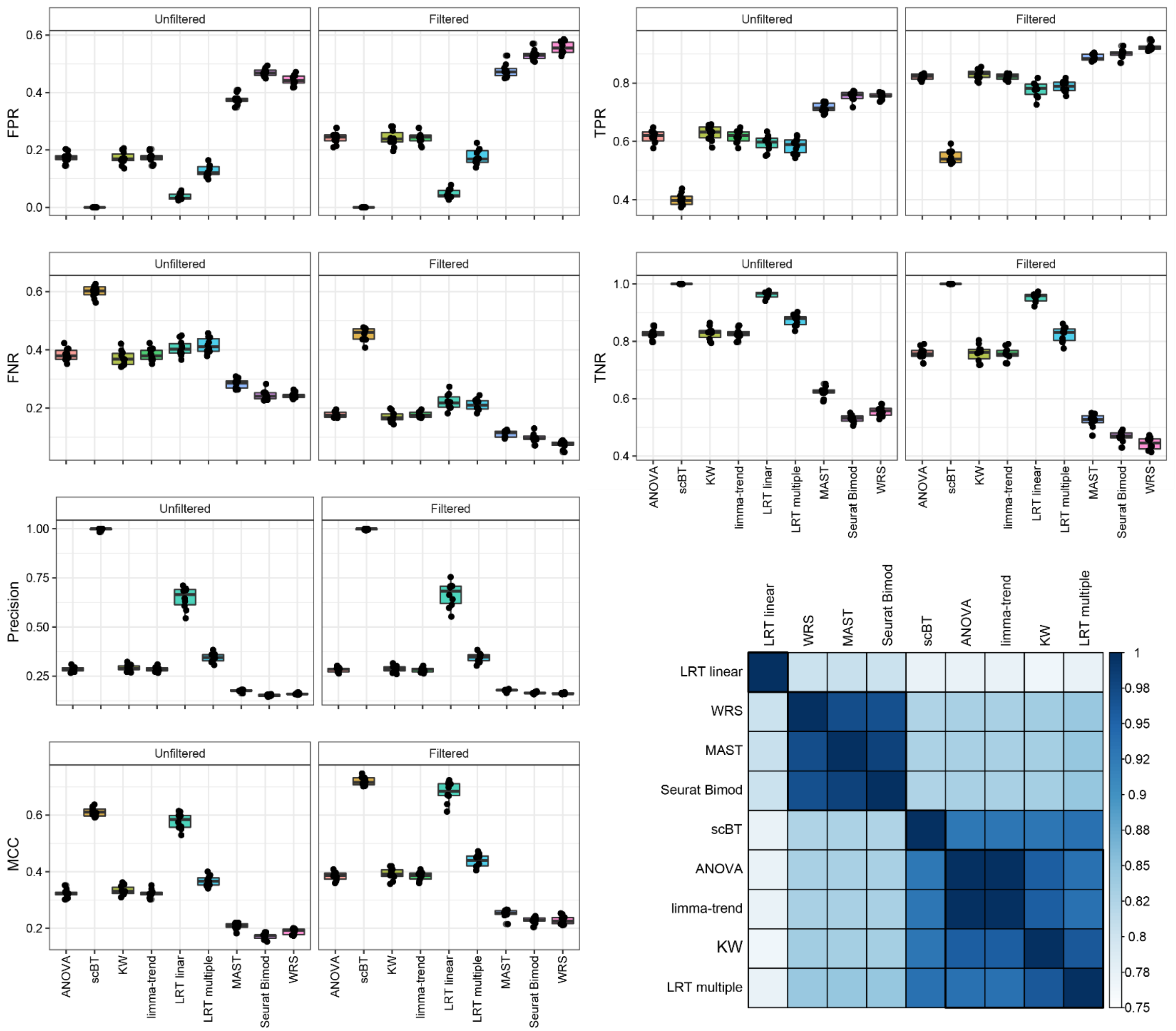

Supplementary Figure 5: Benchmarking metrics of 9 differential expression test methods for simulated dose response data using default initial parameters. A total of 4,500 cells (500 cells per dose group) and 5,000 genes were simulated across 9 dose groups with a probability of being differentially expressed of 10%, of which 50% were repressed. Differential expression fold-change location and scale were 0.8 and 0.4, respectively. Given a ground truth from simulation outputs, false positive rates (FPR), true positive rates (TPR), false negative rates (FNR), true negative rates (TNR), precision, and Matthews correlation coefficient (MCC) were calculated. Points represent median ± minimum to maximum values for 10 replicate simulations. Heat map represents the area under the concordance curve (AUCC) of the 100 most significant gene expression changes (lowest *P*-values) calculated for each pairwise comparison and clustered based on the similarity of the scores. Box and whisker plots represent median and 25th and 75th percentile, and minimum and maximum values for 10 replicate simulations.

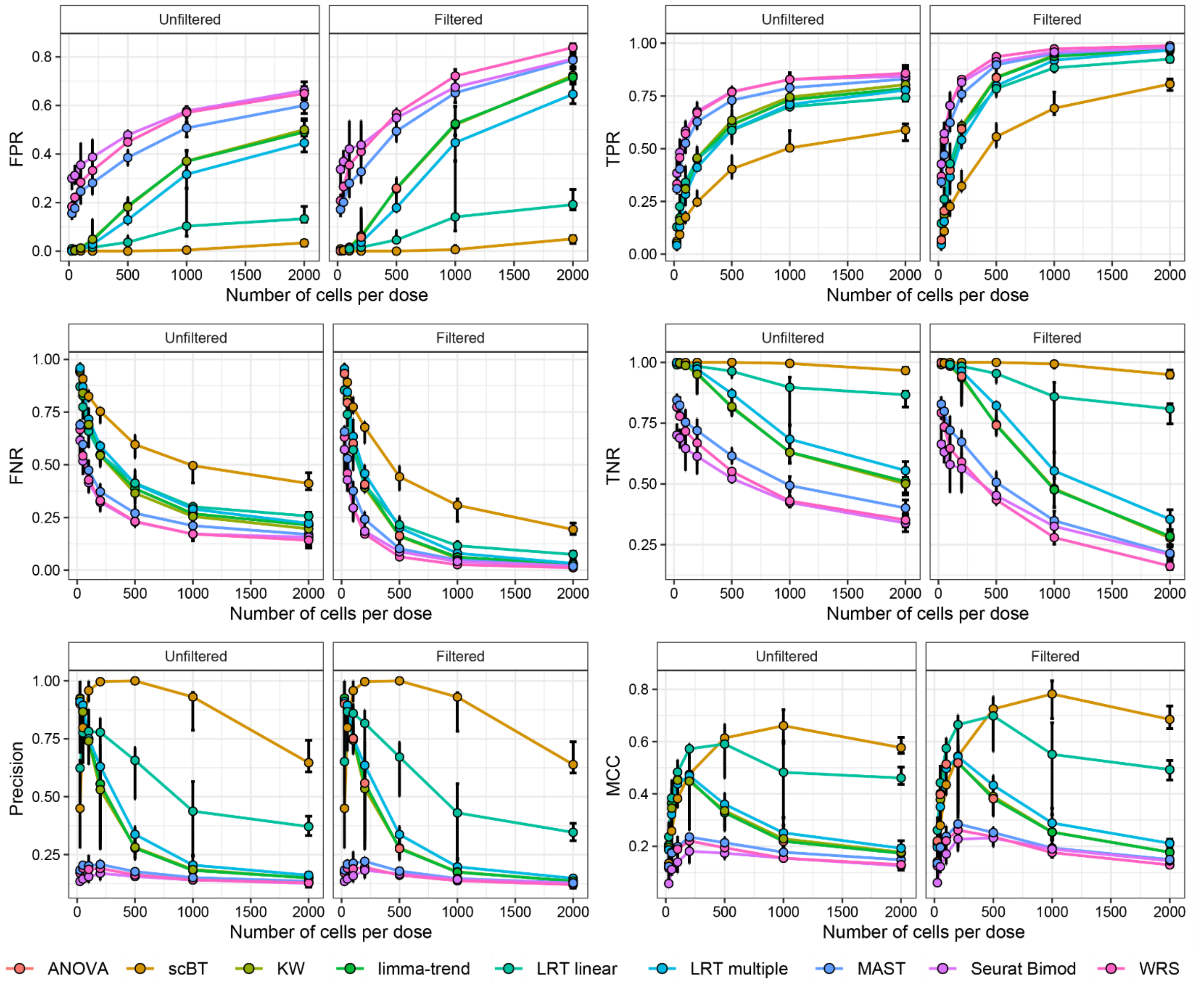

Supplementary Figure 6: Benchmarking metrics of 9 differential expression test methods for simulated dose response data with varying cell abundances. 5,000 genes were simulated across 9 dose groups with a probability of being differentially expressed of 10%, of which 50% were repressed. Differential expression fold-change location and scale were 0.8 and 0.4, respectively. Given a ground truth from simulation outputs, false positive rates (FPR), true positive rates (TPR), false negative rates (FNR), true negative rates (TNR), precision, and Matthews correlation coefficient (MCC) were calculated. Points represent median ± minimum to maximum values for 10 replicate simulations.

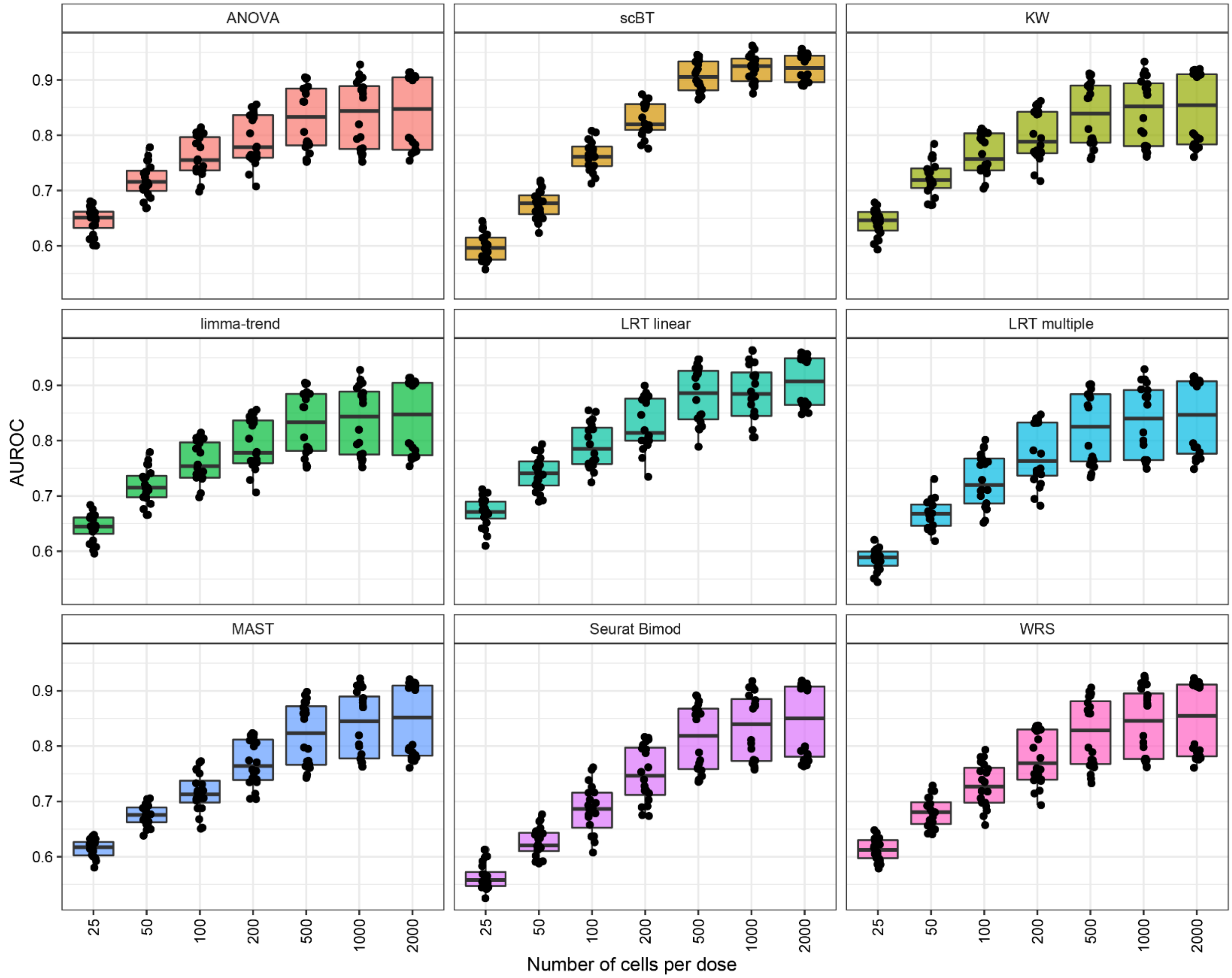

Supplementary Figure 7: Area under the receiver-operating curve (AUROC) of 9 differential expression test methods for simulated dose response data with varying cell abundances. 5,000 genes were simulated across 9 dose groups with a probability of being differentially expressed of 10%, of which 50% were repressed. Differential expression fold-change location and scale were 0.8 and 0.4, respectively. Box and whisker plots represent median and 25th and 75th percentile, and minimum and maximum values for 10 replicate simulations.

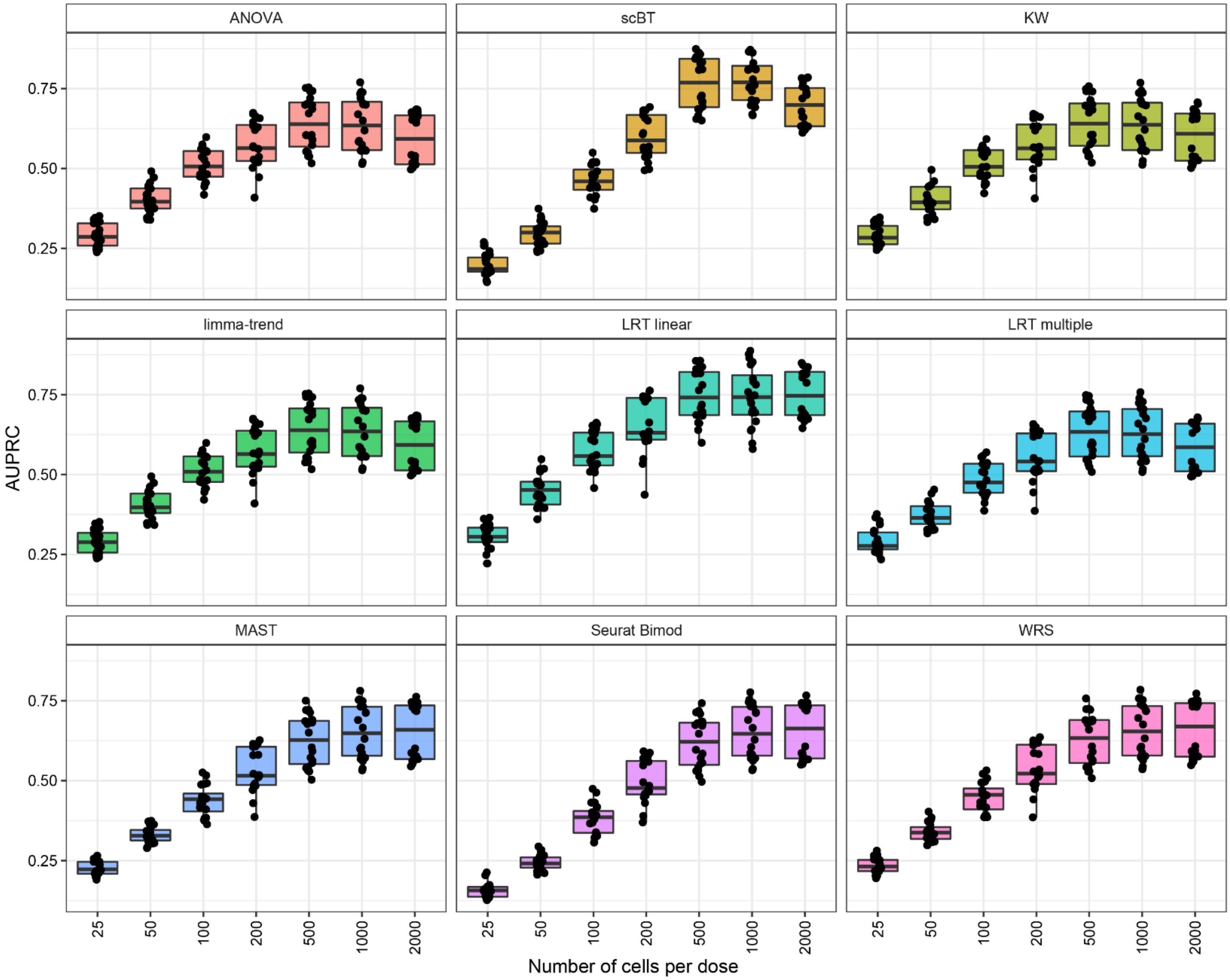

Supplementary Figure 8: Area under the precision-recall curve (AUPRC) of 9 differential expression test methods for simulated dose response data with varying number of cell abundances. 5,000 genes were simulated across 9 dose groups with a probability of being differentially expressed of 10%, of which 50% were repressed. Differential expression fold-change location and scale were 0.8 and 0.4, respectively. Box and whisker plots represent median and 25th and 75th percentile, and minimum and maximum values for 10 replicate simulations.

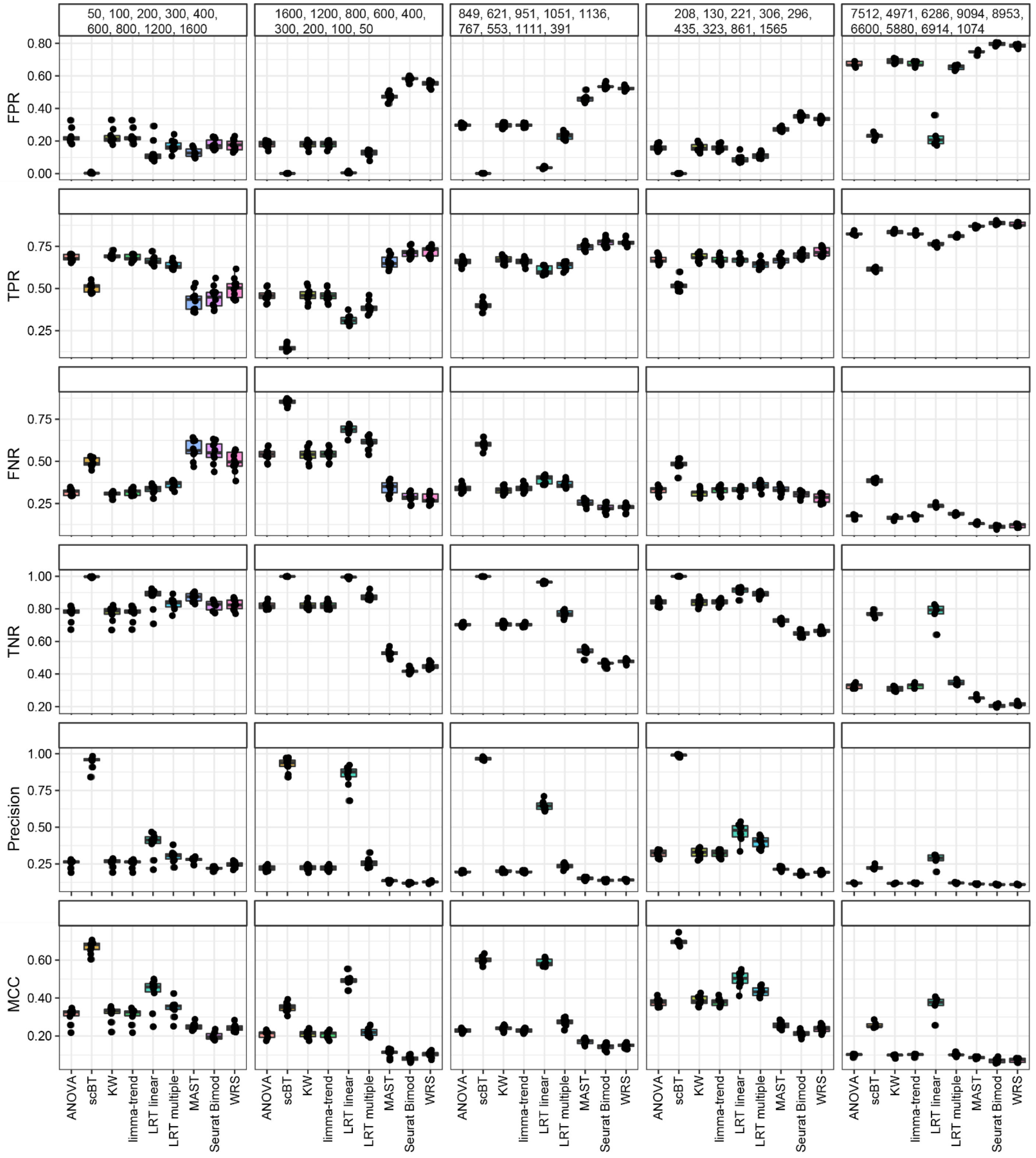

Supplementary Figure 9: Benchmarking metrics of 9 differential expression test methods for simulated dose response data with varying number of cells per dose group. 5,000 genes were simulated across 9 dose groups with a probability of being differentially expressed of 10%, of which 50% were repressed. Differential expression fold-change location and scale were 0.8 and 0.4, respectively. Given a ground truth from simulation outputs, false positive rates (FPR), true positive rates (TPR), false negative rates (FNR), true negative rates (TNR), precision, and Matthews correlation coefficient (MCC) were calculated. Box and whisker plots represent median and 25th and 75th percentile, and minimum and maximum values for 10 replicate simulations.

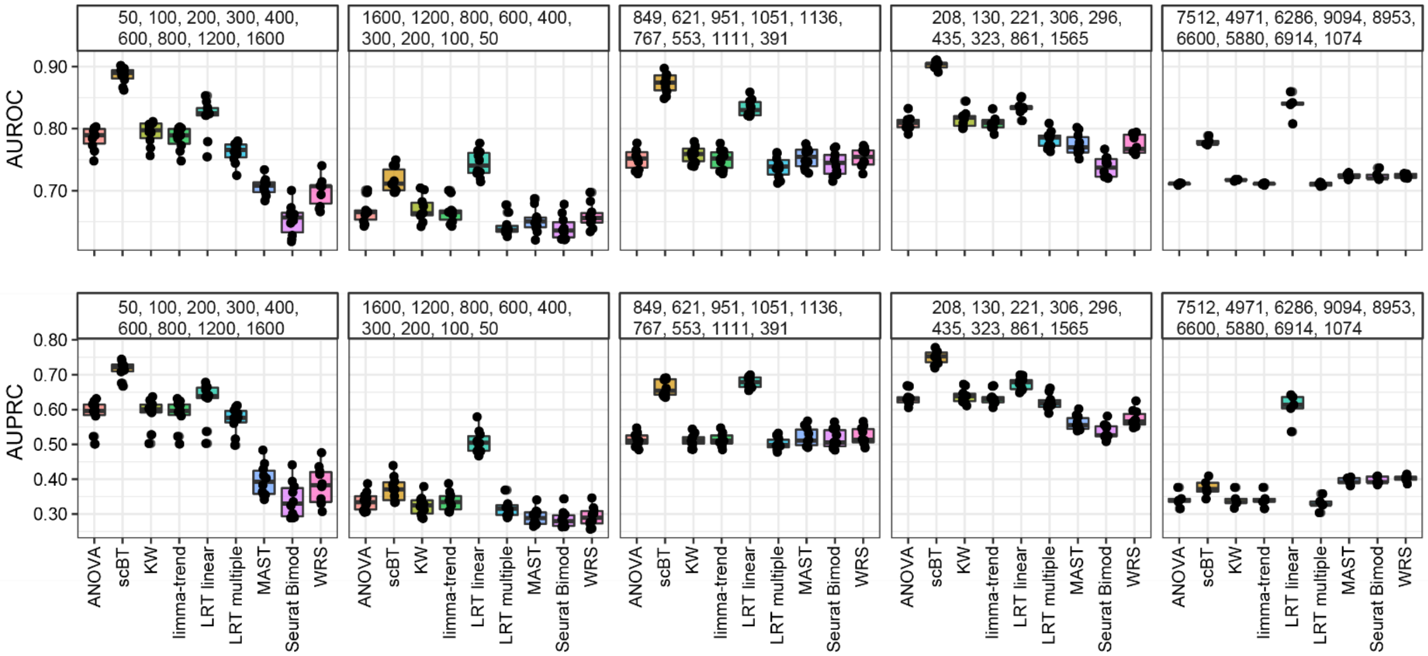

Supplementary Figure 10: Area under the receiver-operating curve (AUROC) and area under the precision-recall curve (AUPRC) of 9 differential expression test methods for simulated dose response data with varying number of cells per dose group. 5,000 genes were simulated across 9 dose groups with a probability of being differentially expressed of 10%, of which 50% were repressed. Box and whisker plots represent median and 25th and 75th percentile, and minimum and maximum values for 10 replicate simulations.

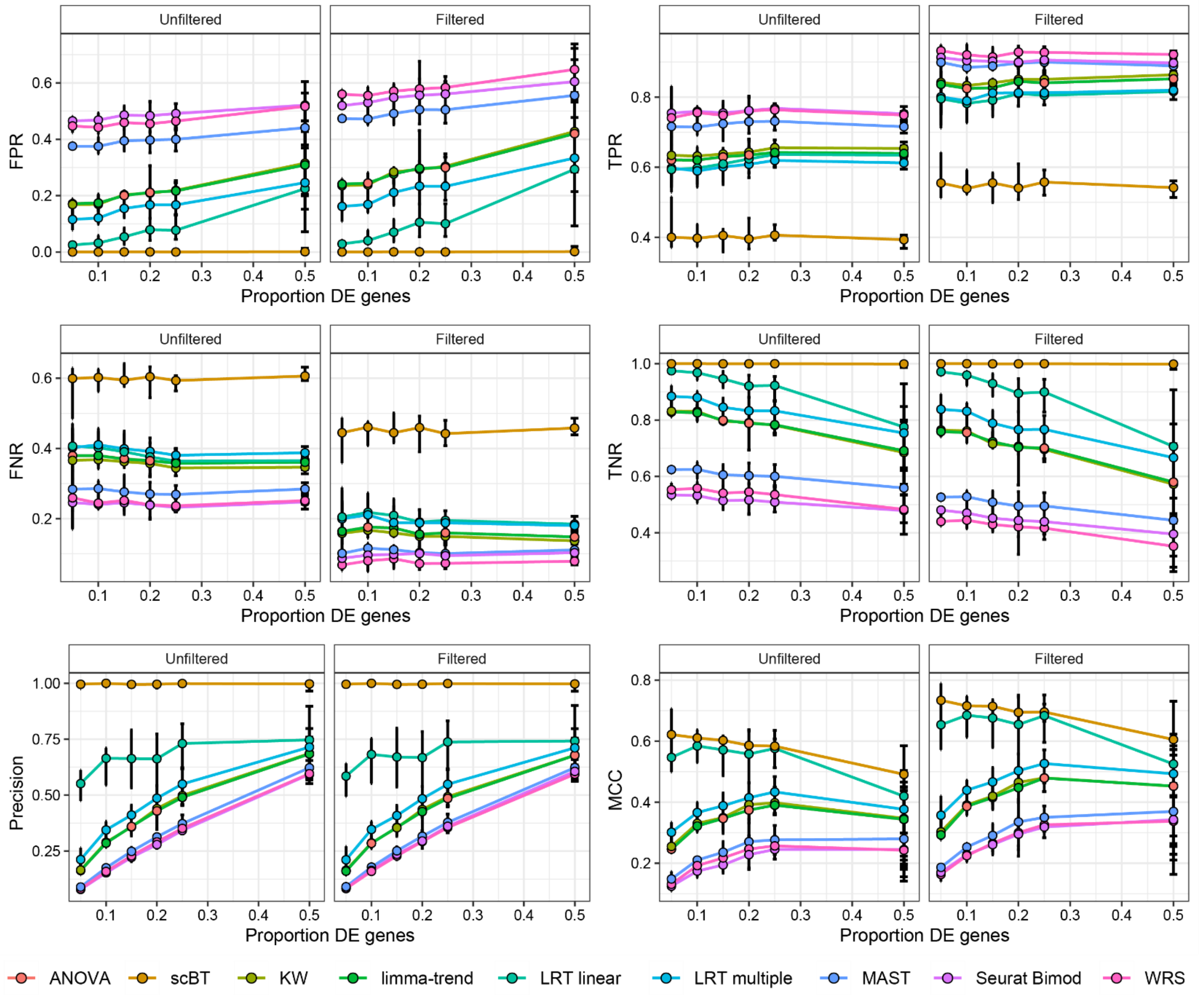

Supplementary Figure 11: Benchmarking metrics of 9 differential expression test methods for simulated dose response data with varying differential expression probabilities. A total of 4,500 cells (500 cells per group) and 5,000 genes were simulated across 9 dose groups with a 50% probability of being repressed. Differential expression fold-change location and scale were 0.8 and 0.4, respectively. Given a ground truth from simulation outputs, false positive rates (FPR), true positive rates (TPR), false negative rates (FNR), true negative rates (TNR), precision, and Matthews correlation coefficient (MCC) were calculated. Points represent median ± minimum to maximum values for 10 replicate simulations.

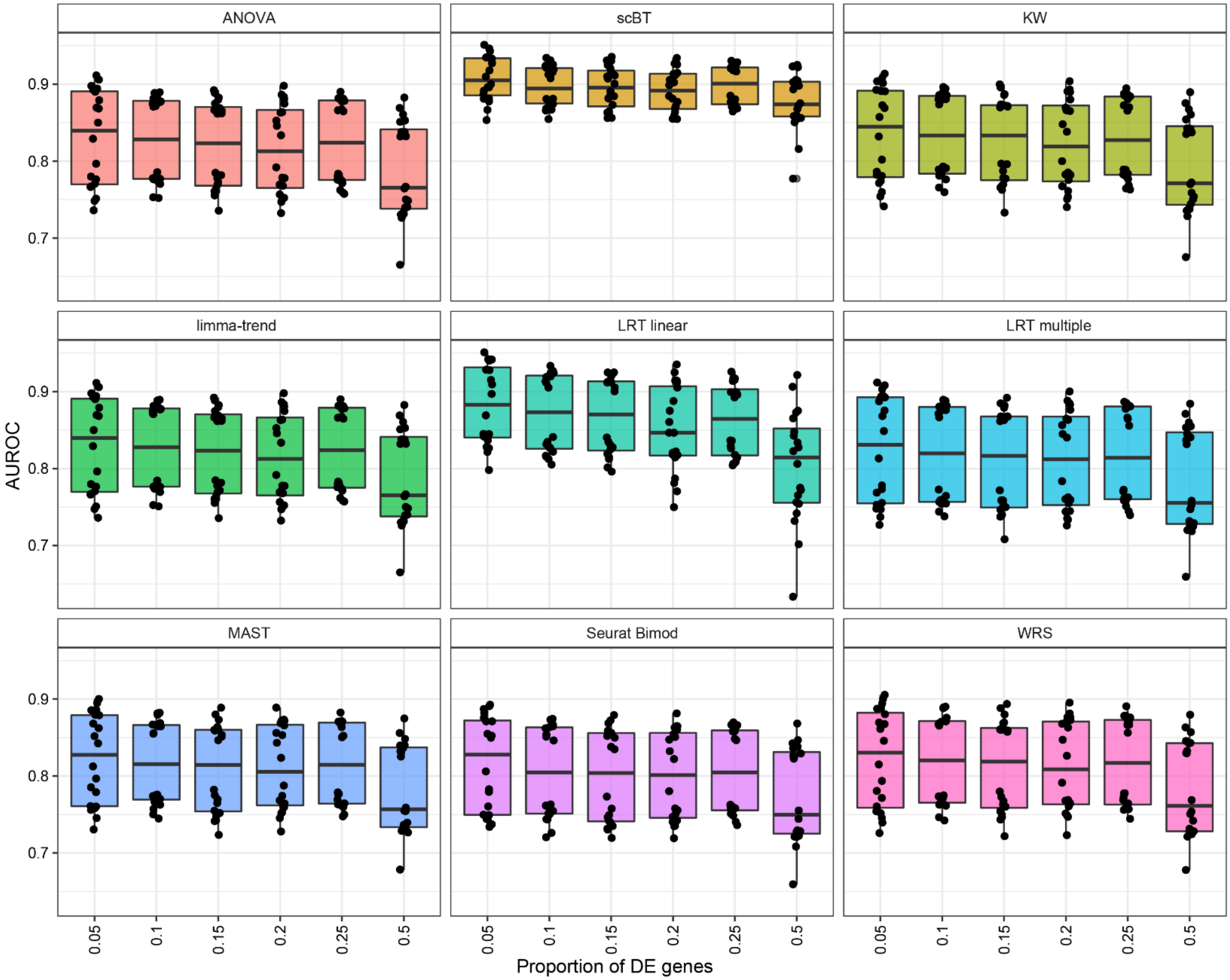

Supplementary Figure 12: Area under the receiver-operating curve (AUROC) of 9 differential expression test methods for simulated dose response data with varying differential expression probabilities. A total of 4,500 cells (500 cells per group) and 5,000 genes were simulated across 9 dose groups with a 50% probability of being repressed. Differential expression fold-change location and scale were 0.8 and 0.4, respectively. Box and whisker plots represent median and 25th and 75th percentile, and minimum and maximum values for 10 replicate simulations.

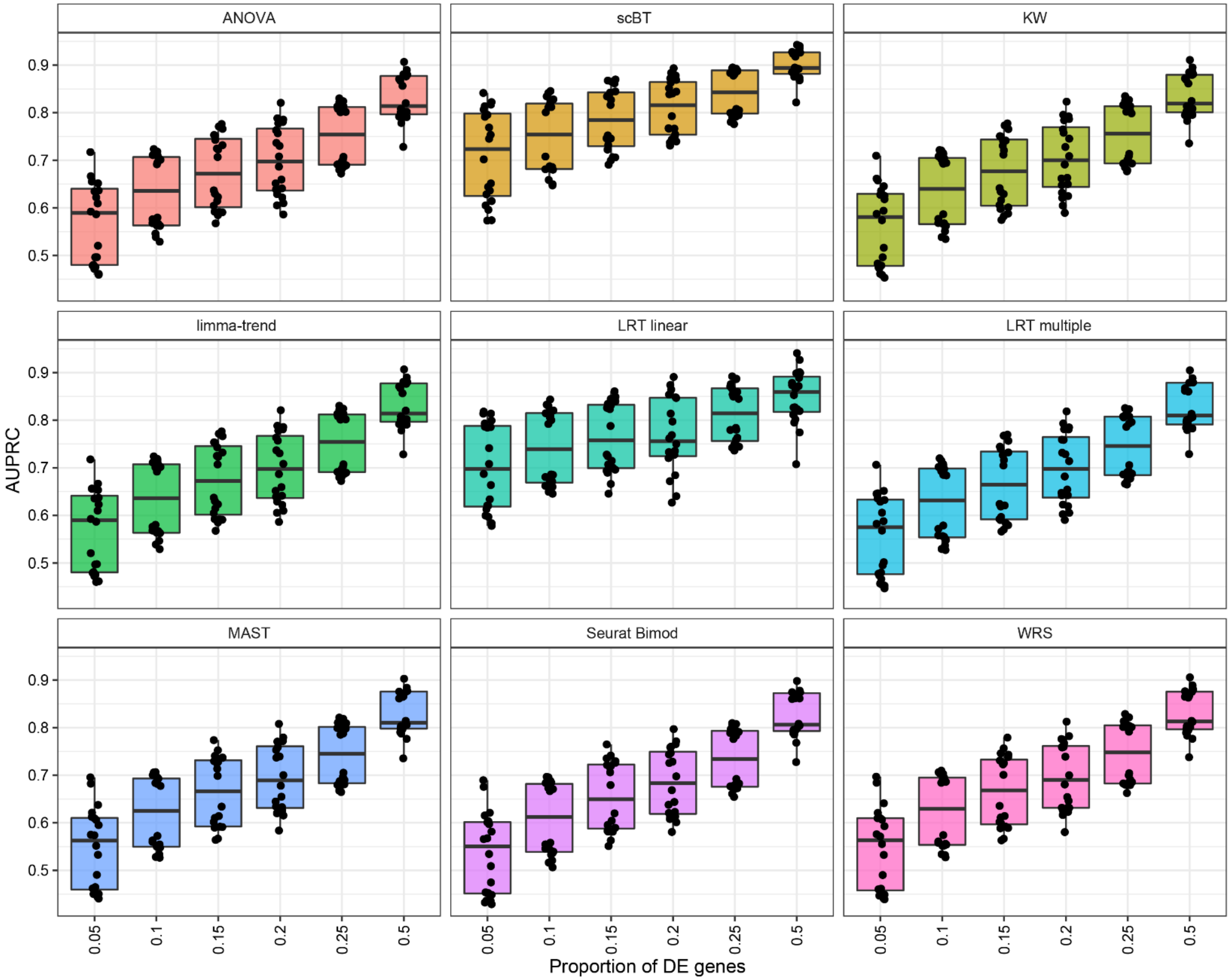

Supplementary Figure 13: Area under the receiver-operating curve (AUPRC) of 9 differential expression test methods for simulated dose response data with varying differential expression probabilities. A total of 4,500 cells (500 cells per group) and 5,000 genes were simulated across 9 dose groups with a 50% probability of being repressed. Differential expression fold-change location and scale were 0.8 and 0.4, respectively. Box and whisker plots represent median and 25th and 75th percentile, and minimum and maximum values for 10 replicate simulations.

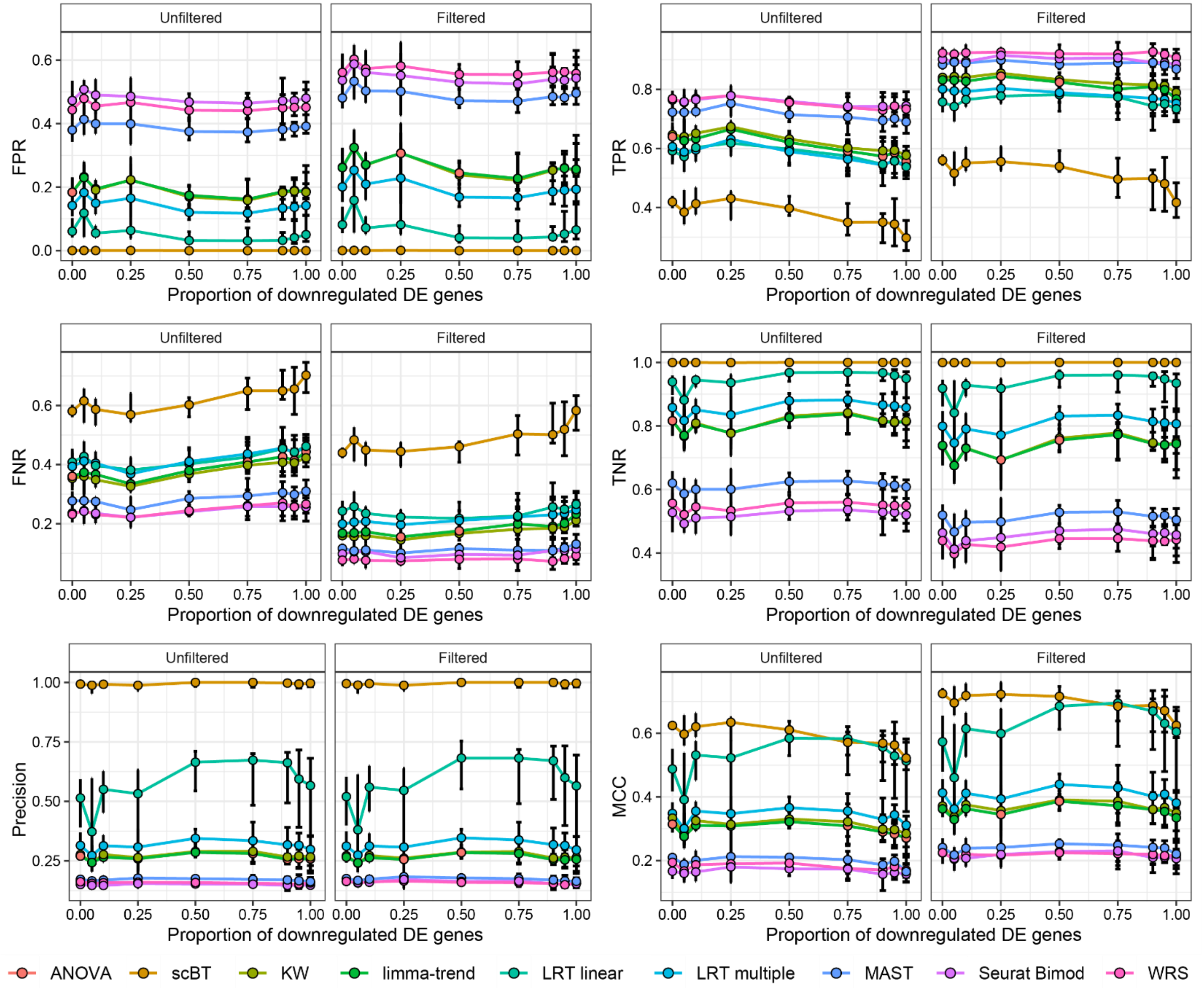

Supplementary Figure 14: Benchmarking metrics of 9 differential expression test methods for simulated dose response data with varying probability of repressed genes. A total of 4,500 cells (500 cells per group) and 5,000 genes were simulated across 9 dose groups with a 10% probability of differential expression. Differential expression fold-change location and scale were 0.8 and 0.4, respectively. Given a ground truth from simulation outputs, false positive rates (FPR), true positive rates (TPR), false negative rates (FNR), true negative rates (TNR), precision, and Matthews correlation coefficient (MCC) were calculated. Points represent median ± minimum to maximum values for 10 replicate simulations.

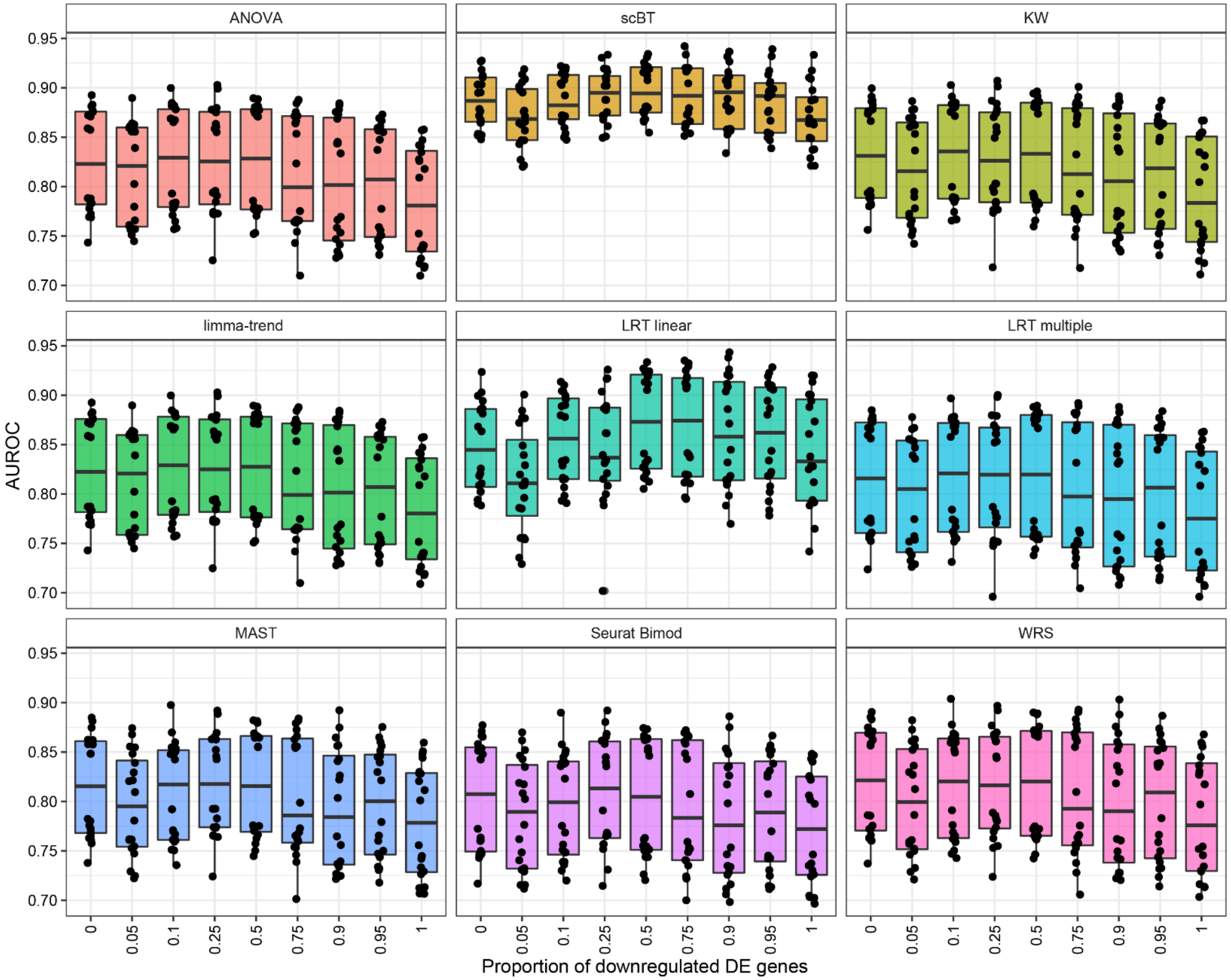

Supplementary Figure 15: Area under the receiver-operating curve (AUROC) of 9 differential expression test methods for simulated dose response data with varying probability of repressed genes. A total of 4,500 cells (500 cells per group) and 5,000 genes were simulated across 9 dose groups with a 10% probability of differential expression. Differential expression fold-change location and scale were 0.8 and 0.4, respectively. Box and whisker plots represent median and 25th and 75th percentile, and minimum and maximum values for 10 replicate simulations.

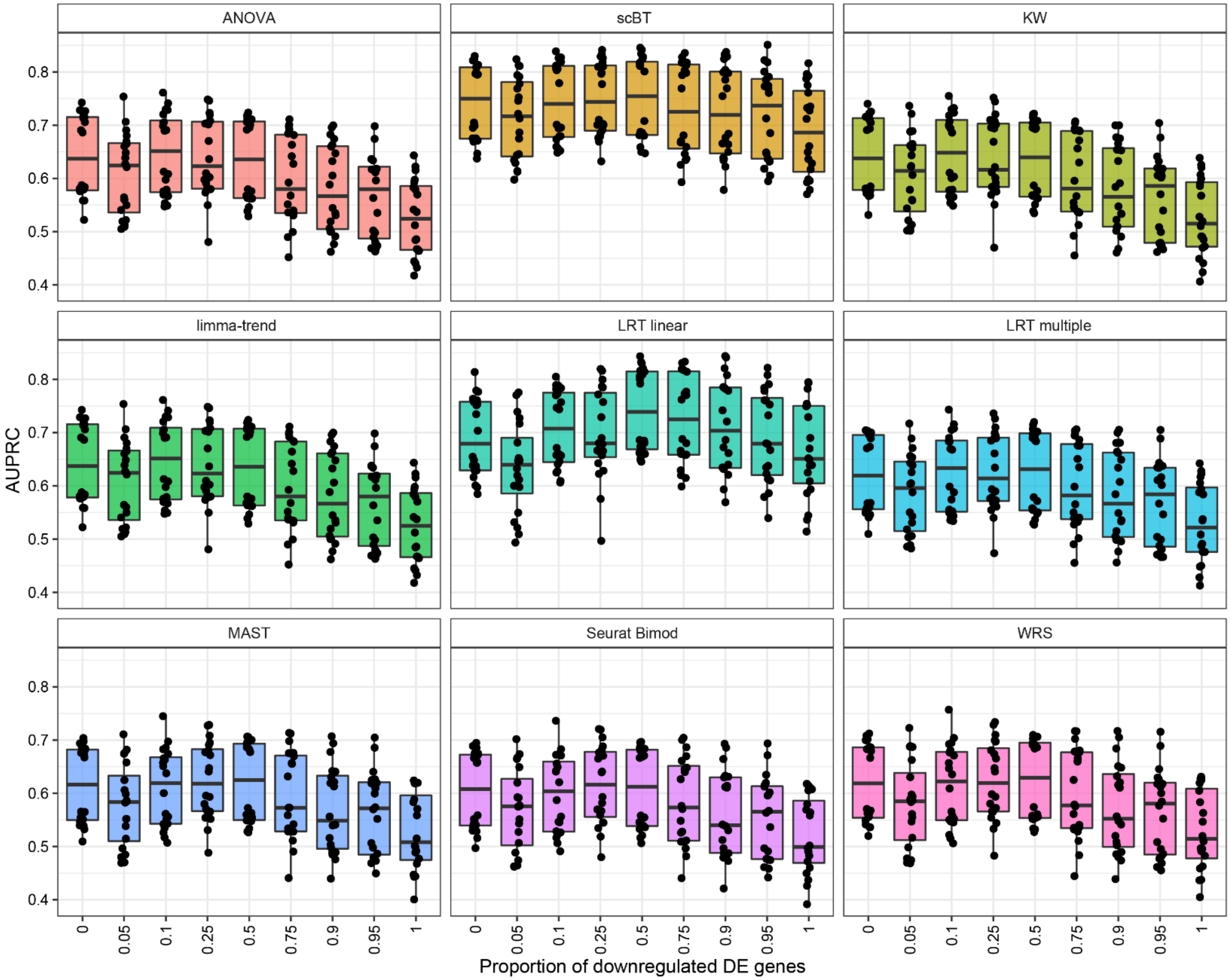

Supplementary Figure 16: Area under the receiver-operating curve (AUPRC) of 9 differential expression test methods for simulated dose response data with varying probability of repressed genes. A total of 4,500 cells (500 cells per group) and 5,000 genes were simulated across 9 dose groups with a 10% probability of differential expression. Differential expression fold-change location and scale were 0.8 and 0.4, respectively. Box and whisker plots represent median and 25th and 75th percentile, and minimum and maximum values for 10 replicate simulations.

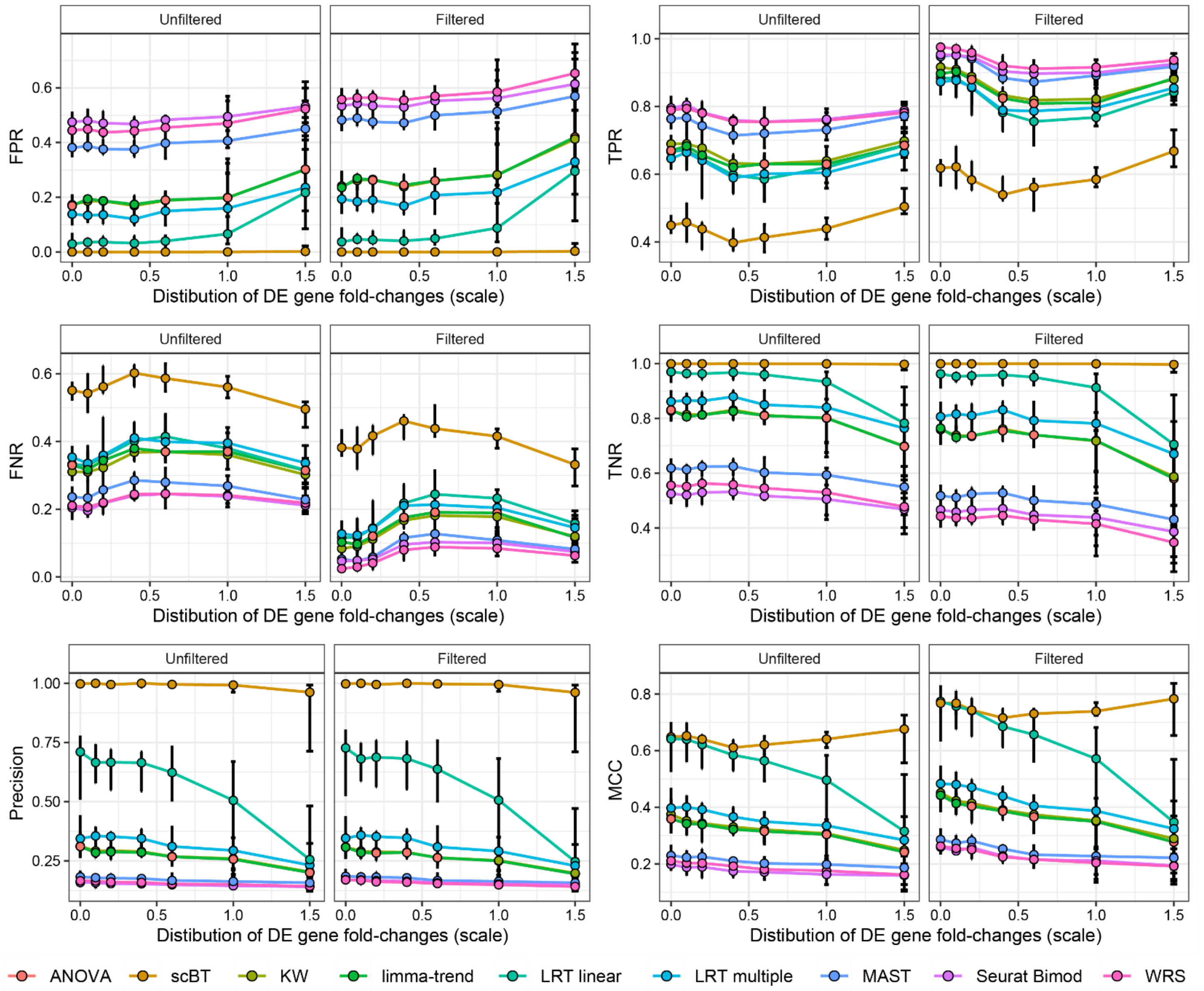

Supplementary Figure 17: Benchmarking metrics of 9 differential expression test methods for simulated dose response data with varying scale of differentially expressed genes. A total of 4,500 cells (500 cells per group) and 5,000 genes were simulated across 9 dose groups with a probability of being differentially expressed of 10%, of which 50% were repressed. Differential expression fold-change location was 0.8. Given a ground truth from simulation outputs, false positive rates (FPR), true positive rates (TPR), false negative rates (FNR), true negative rates (TNR), precision, and Matthews correlation coefficient (MCC) were calculated. Points represent median ± minimum to maximum values for 10 replicate simulations.

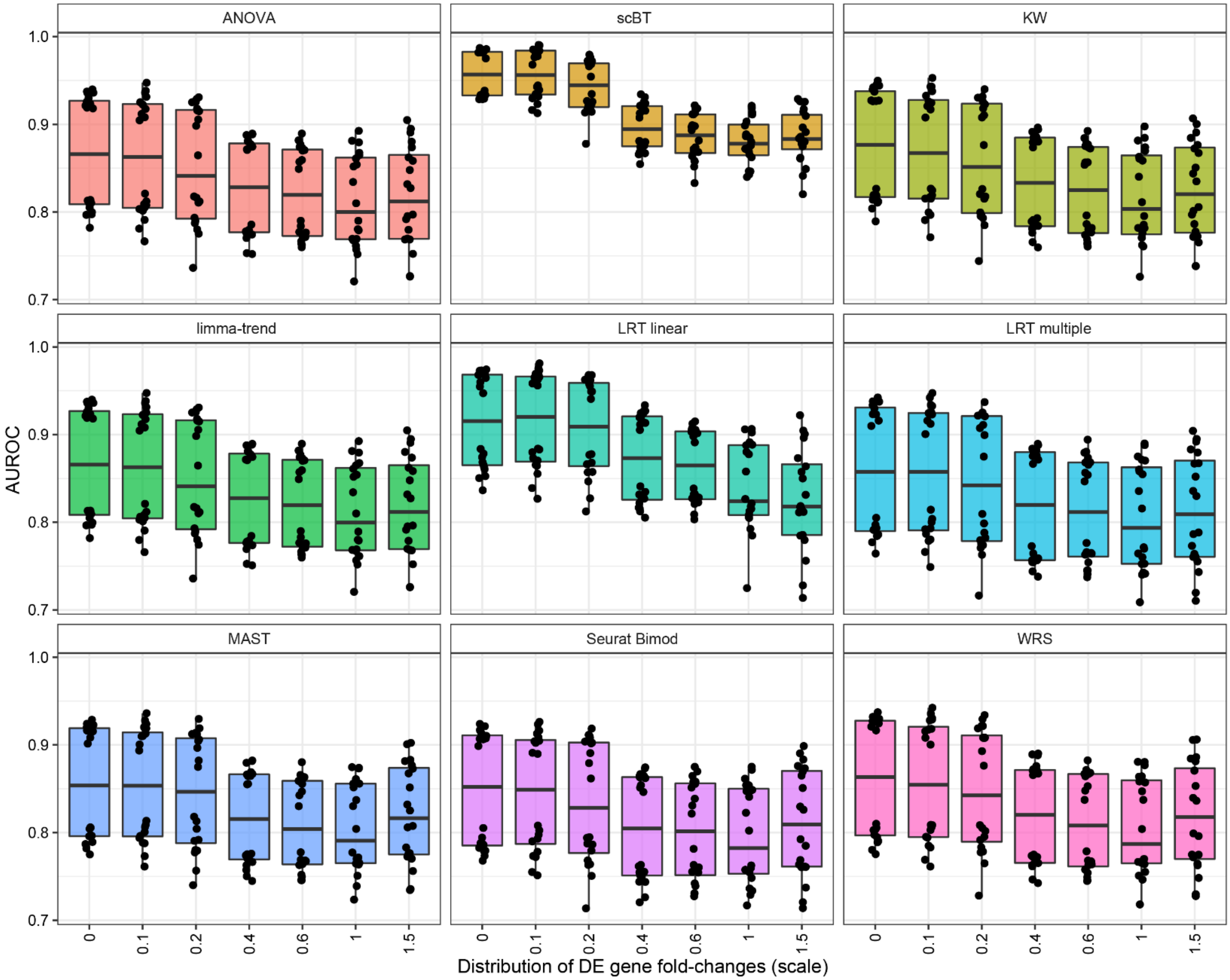

Supplementary Figure 18: Area under the receiver-operating curve (AUROC) of 9 differential expression test methods for simulated dose response data with varying scale of differentially expressed genes. A total of 4,500 cells (500 cells per group) and 5,000 genes were simulated across 9 dose groups with a probability of being differentially expressed of 10%, 50% of which were repressed. Differential expression fold-change location was 0.8. Box and whisker plots represent median and 25th and 75th percentile, and minimum and maximum values for 10 replicate simulations.

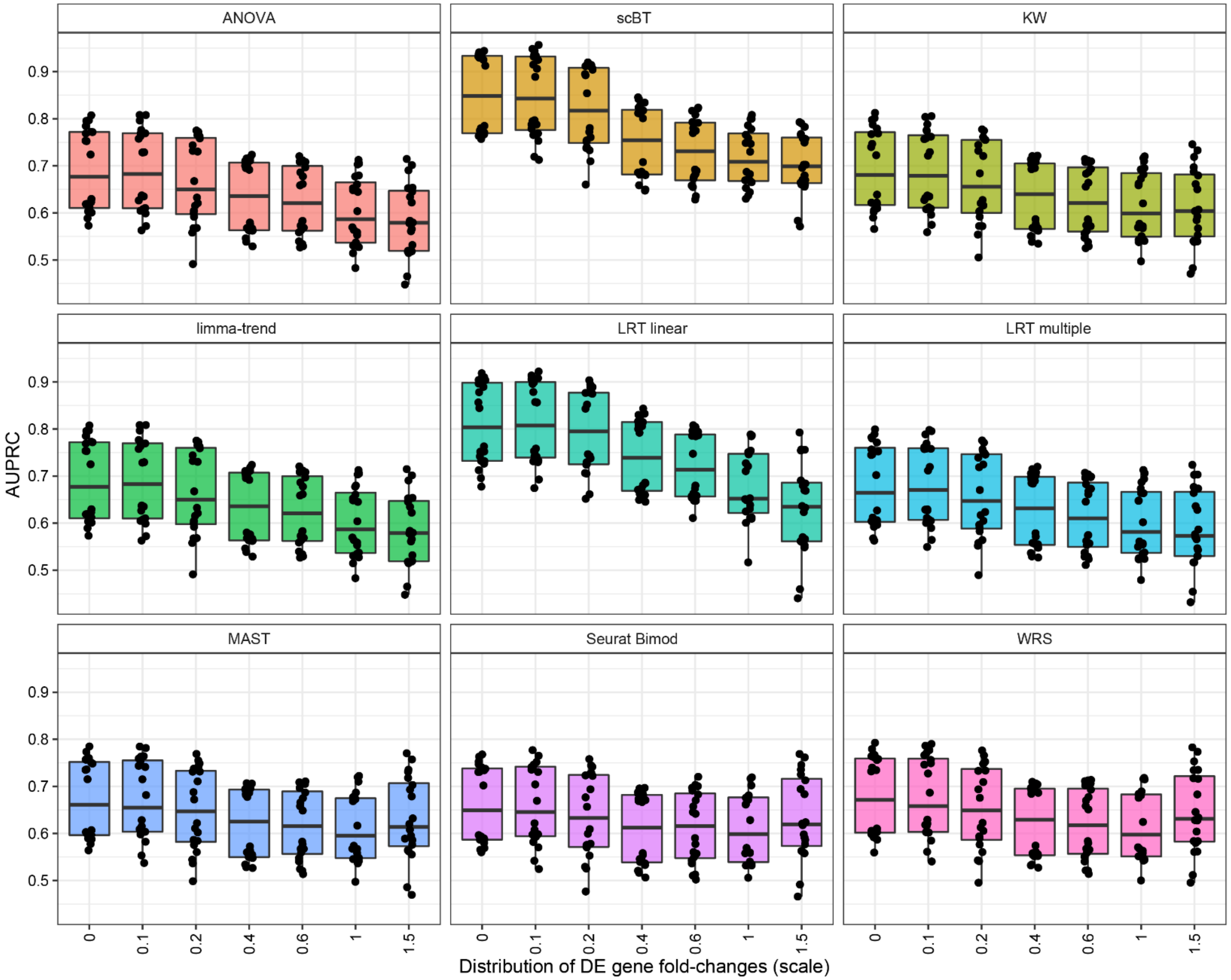

Supplementary Figure 19: Area under the precision-recall curve (AUPRC) of 9 differential expression test methods for simulated dose response data with varying scale of differentially expressed genes. A total of 4,500 cells (500 cells per group) and 5,000 genes were simulated across 9 dose groups with a probability of being differentially expressed of 10%, of which 50% were repressed. Differential expression fold-change location was 0.8. Box and whisker plots represent median and 25th and 75th percentile, and minimum and maximum values for 10 replicate simulations.

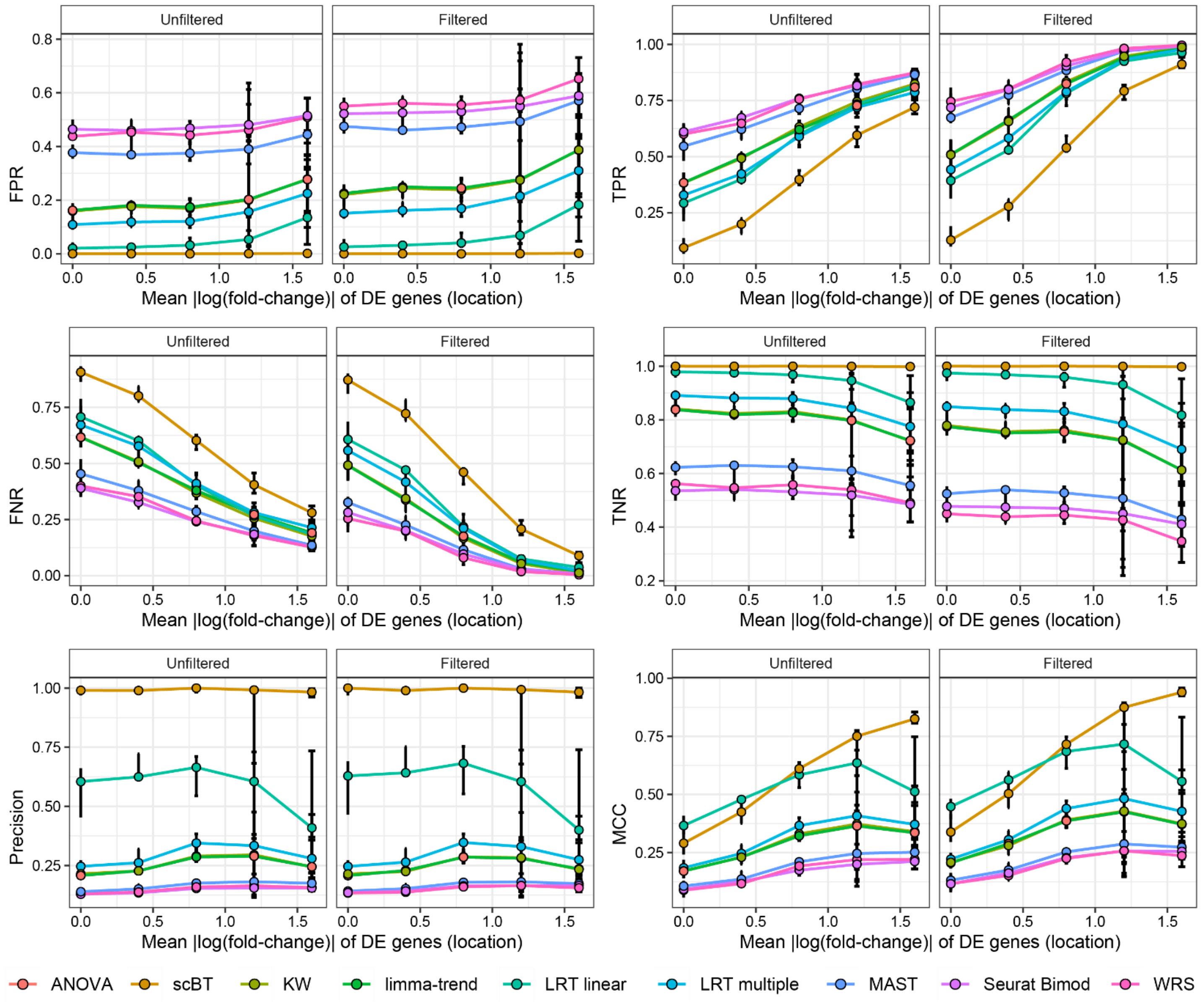

Supplementary Figure 20: Benchmarking metrics of 9 differential expression test methods for simulated dose response data with varying location of differentially expressed genes. A total of 4,500 cells (500 cells per group) and 5,000 genes were simulated across 9 dose groups with a probability of being differentially expressed of 10%, of which 50% were repressed. Differential expression fold-change scale was 0.4. Given a ground truth from simulation outputs, false positive rates (FPR), true positive rates (TPR), false negative rates (FNR), true negative rates (TNR), precision, and Matthews correlation coefficient (MCC) were calculated. Points represent median ± minimum to maximum values for 10 replicate simulations.

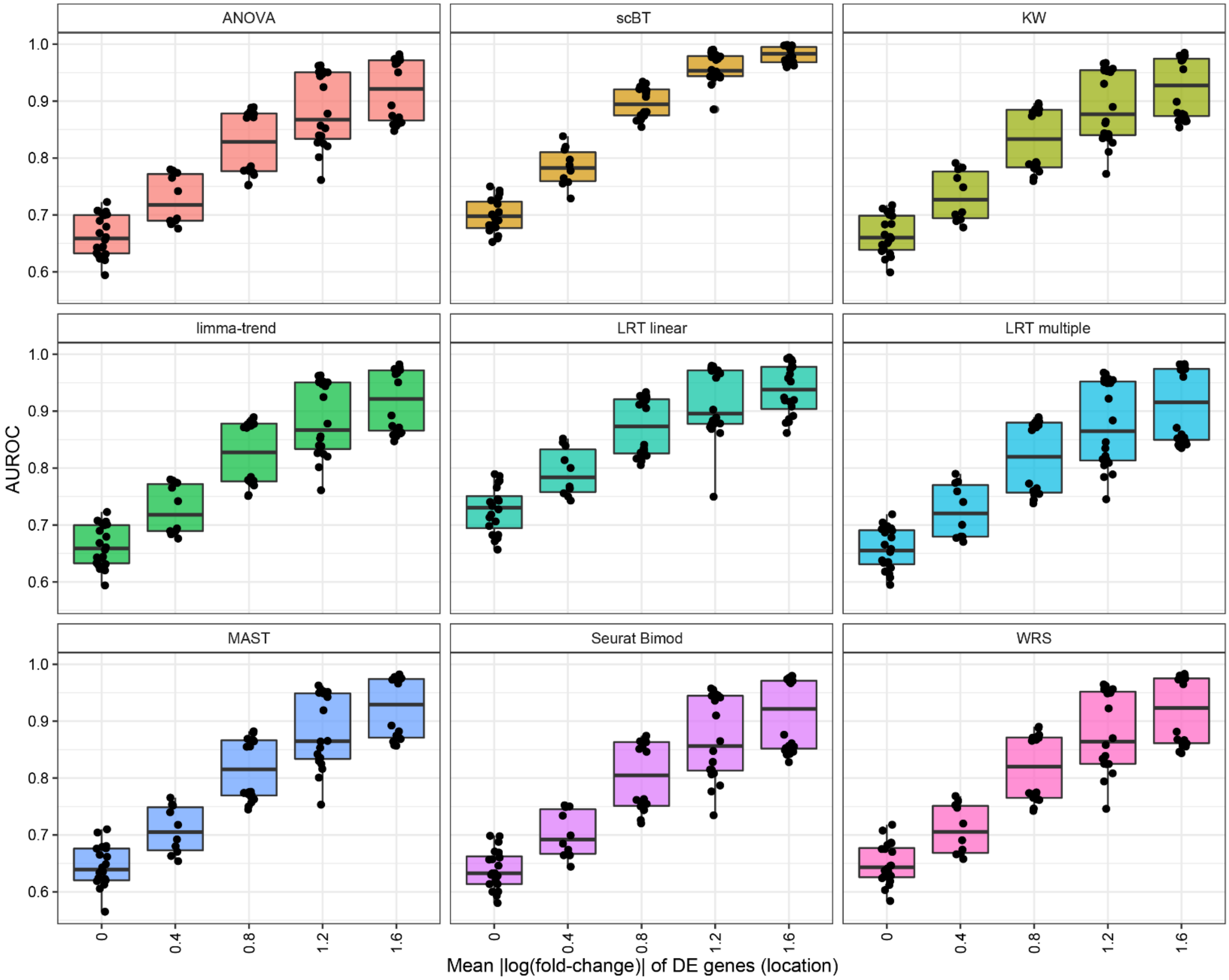

Supplementary Figure 21: Area under the receiver-operating curve (AUROC) of 9 differential expression test methods for simulated dose response data with varying location of differentially expressed genes. A total of 4,500 cells (500 cells per group) and 5,000 genes were simulated across 9 dose groups with a probability of being differentially expressed of 10%, of which 50% were repressed. Differential expression fold-change scale was 0.4. Box and whisker plots represent median and 25th and 75th percentile, and minimum and maximum values for 10 replicate simulations.

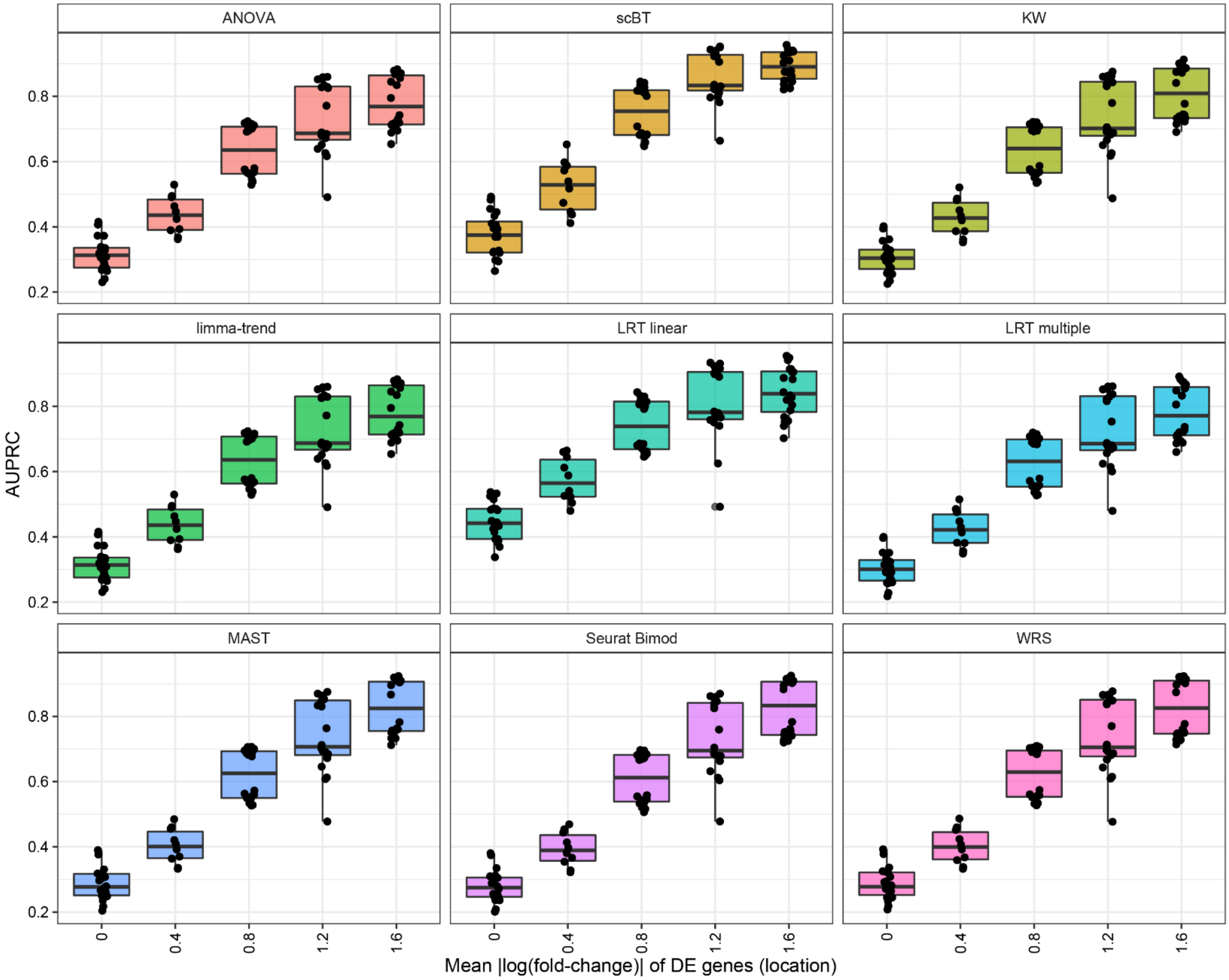

Supplementary Figure 22: Area under the precision-recall curve (AUPRC) of 9 differential expression test methods for simulated dose response data with varying location of differentially expressed genes. A total of 4,500 cells (500 cells per group) and 5,000 genes were simulated across 9 dose groups with a probability of being differentially expressed of 10%, of which 50% were repressed. Differential expression fold-change scale was 0.4. Box and whisker plots represent median and 25th and 75th percentile, and minimum and maximum values for 10 replicate simulations.

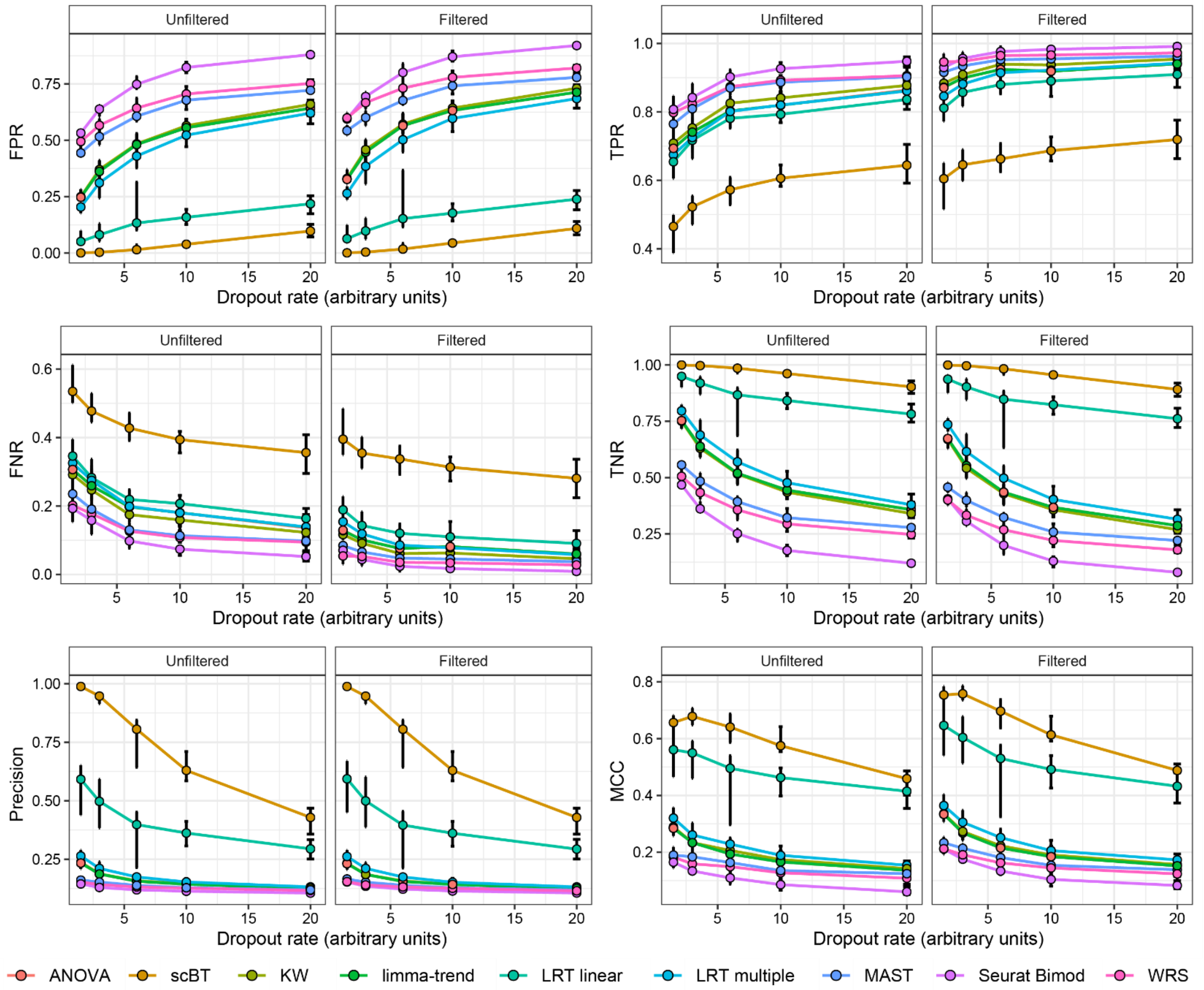

Supplementary Figure 23: Benchmarking metrics of 9 differential expression test methods for simulated dose response data with varying relationship between mean expression and percent zeroes. A total of 4,500 cells (500 cells per group) and 5,000 genes were simulated across 9 dose groups with a probability of being differentially expressed of 10%, of which 50% were downregulated. Differential expression fold-change location and scale were 0.8 and 0.4, respectively. Given a ground truth from simulation outputs, false positive rates (FPR), true positive rates (TPR), false negative rates (FNR), true negative rates (TNR), precision, and Matthews correlation coefficient (MCC) were calculated. Points represent median ± minimum to maximum values for 10 replicate simulations. Dropout rates are calculated as $Percent\ zeroes = a * e^{b*mean\ log\ expression} + t$ where parameters are shown in **Table S2**.

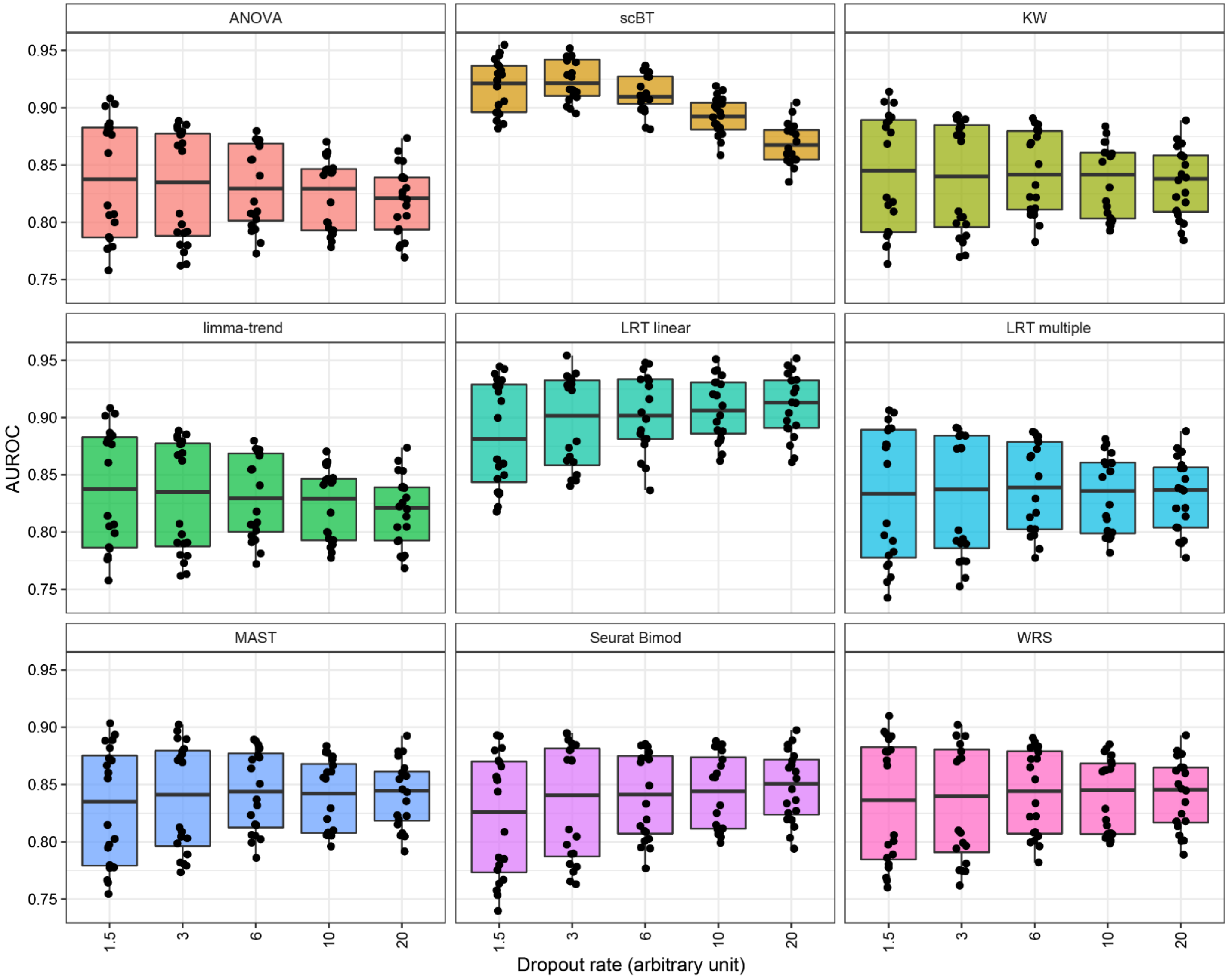

Supplementary Figure 24: Area under the receiver-operating curve (AUROC) of 9 differential expression test methods for simulated dose response data with varying relationship between mean expression and percent zeroes. A total of 4,500 cells (500 cells per group) and 5,000 genes were simulated across 9 dose groups with a probability of being differentially expressed of 10%, of which 50% were repressed. Differential expression fold-change location and scale were 0.8 and 0.4, respectively. Box and whisker plots represent median and 25th and 75th percentile, and minimum and maximum values for 10 replicate simulations. Dropout rates are calculated as $Percent\ zeroes = a * \ e^{b*mean\ log\ expression} + t$ where parameters are shown in **Table S2**.

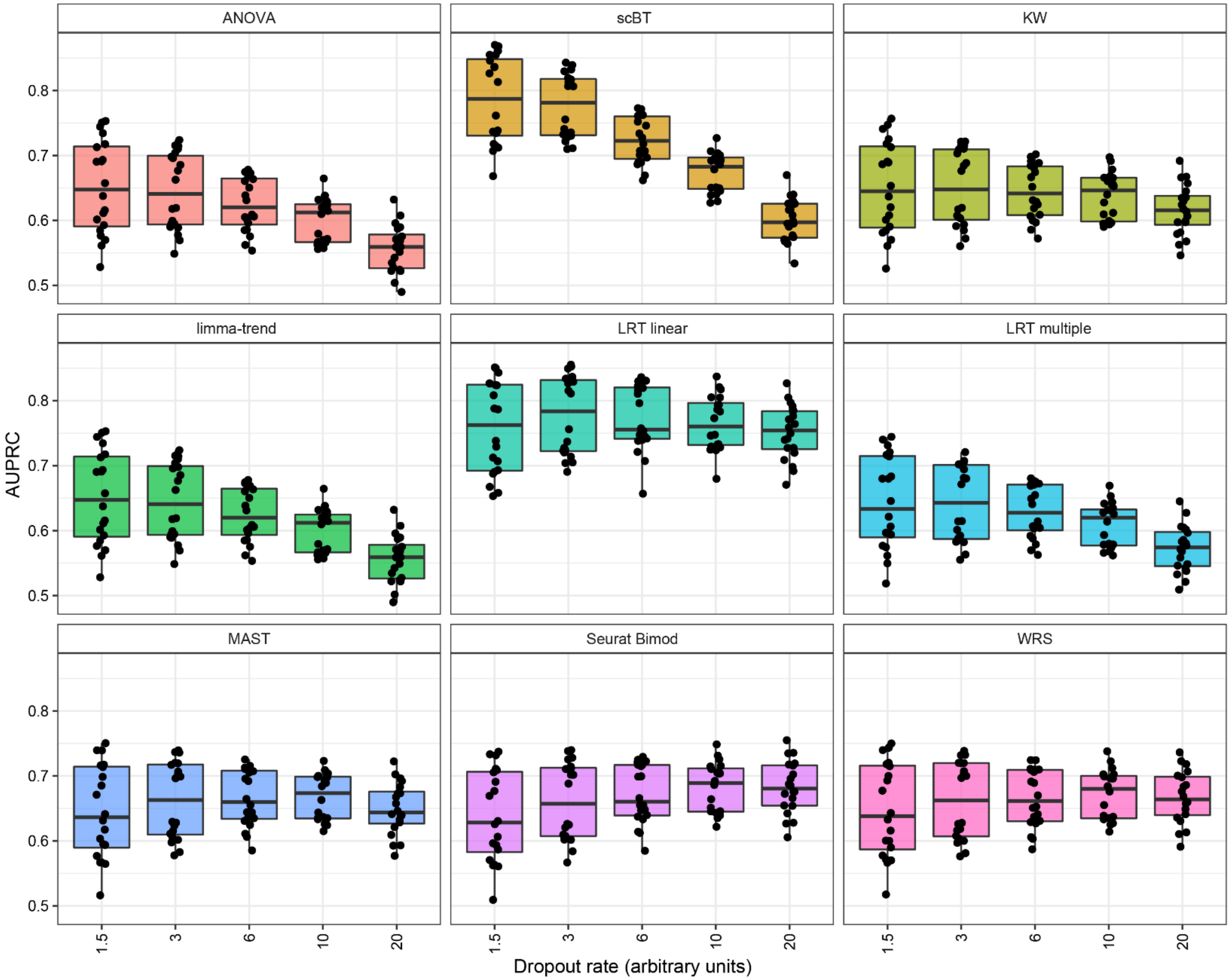

Supplementary Figure 25: Area under the precision-recall curve (AUPRC) of 9 differential expression test methods for simulated dose response data with varying relationship between mean expression and percent zeroes. A total of 4,500 cells (500 cells per group) and 5,000 genes were simulated across 9 dose groups with a probability of being differentially expressed of 10%, of which 50% were repressed. Differential expression fold-change location and scale were 0.8 and 0.4, respectively. Box and whisker plots represent median and 25th and 75th percentile, and minimum and maximum values for 10 replicate simulations. Dropout rates are calculated as $Percent\ zeroes = a * e^{b*mean\ log\ expression} + t$ where parameters are shown in **Table S2**.

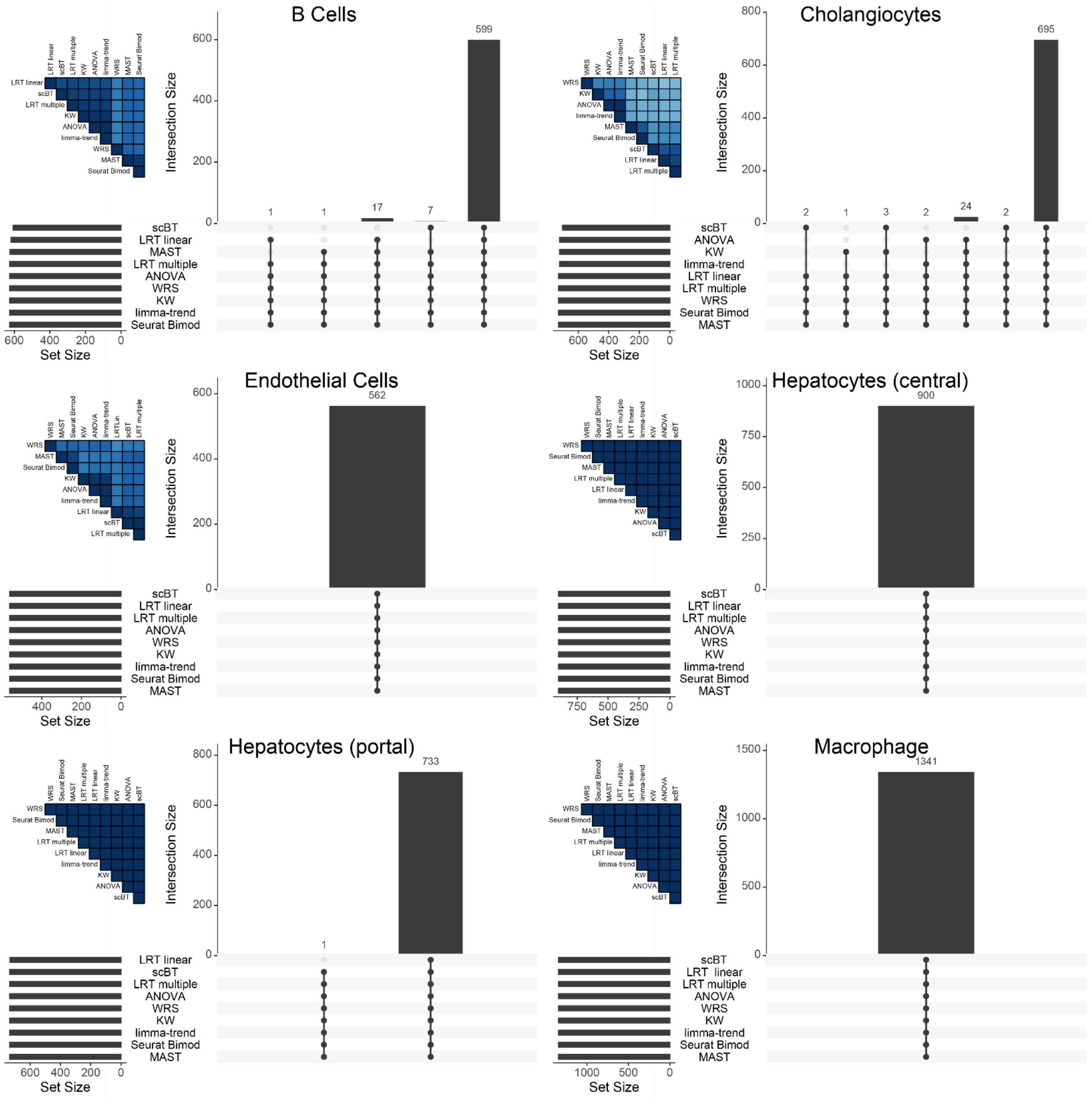

Supplementary Figure 26: Comparison of differential gene expression analysis of hepatic single-nuclei RNA sequencing data from male mice gavaged with sesame oil vehicle control or 0.01 – 30 µg/kg TCDD every 4 days for 28 days. Each panel represents a distinct cell type showing the intersection of differentially expressed genes (vertical bars) for each combination of tests (filled circles) and total number of differentially expressed genes (horizontal bars). Intersect sizes are displayed on top of vertical bars. The tile plot in the upper left represents the area under the concordance curve (AUCC) calculated in the bottom right panel (page 2). A higher score indicates stronger agreement in the lowest 500 ranked adjusted *p*-values. Genes were considered differentially expressed when expressed in at least 5% of cells in any dose group and with a |fold-change| ≥ 1.5.

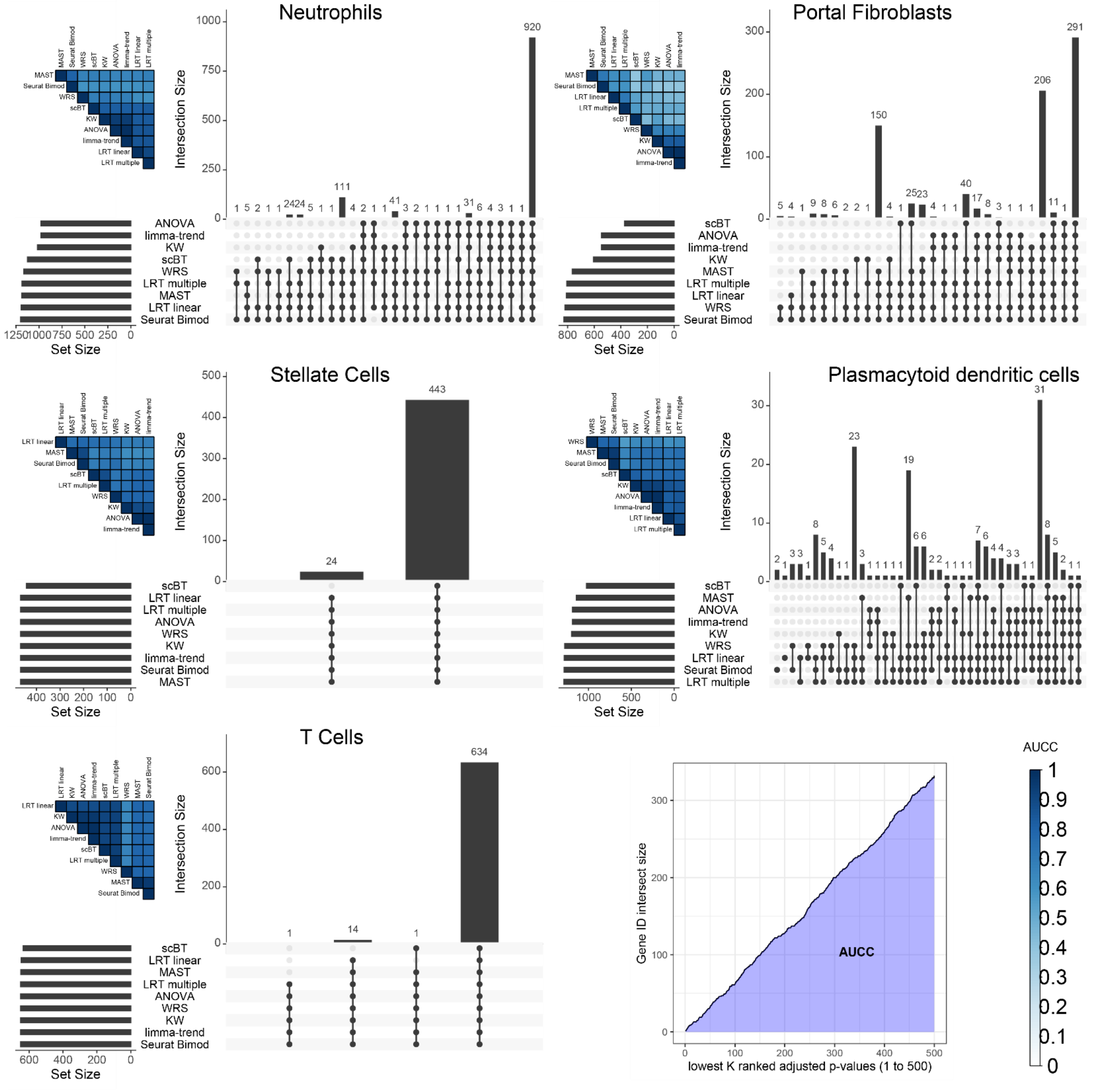

Supplementary Figure 26: (continued)

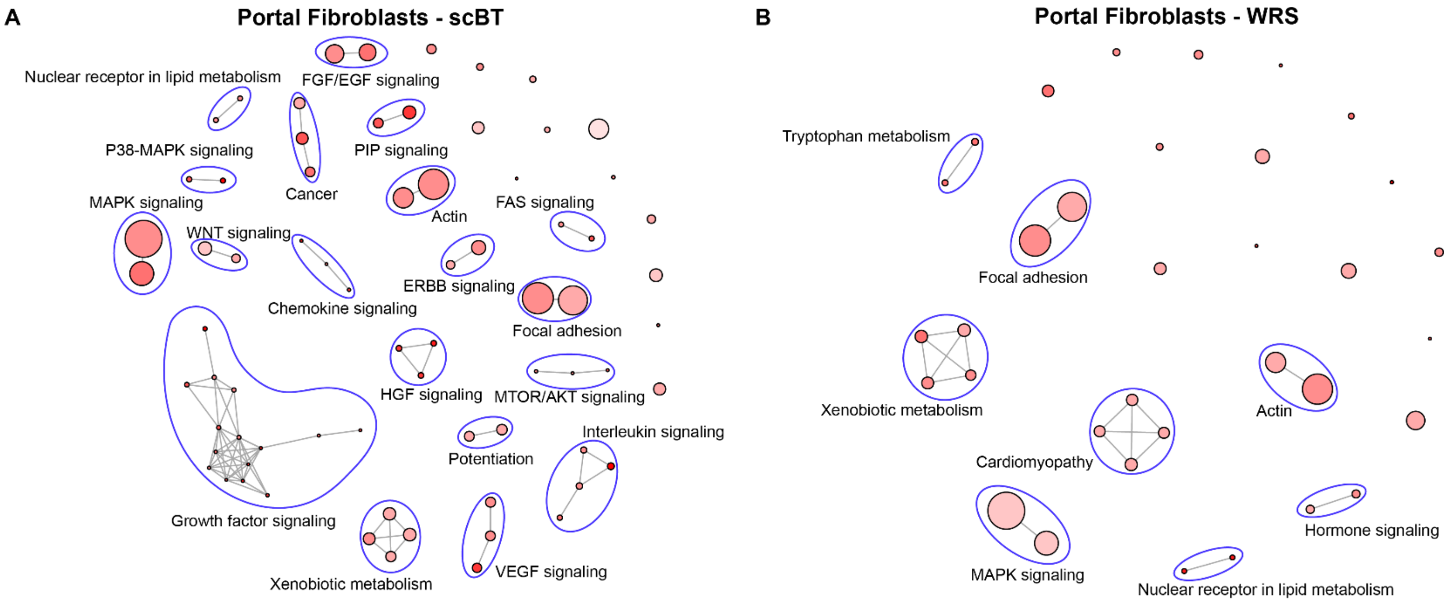

Supplementary Figure 27: Network of significantly enriched gene sets based on DGEA analysis of portal fibroblasts single-nuclei RNA sequencing data from male mice gavaged with sesame oil vehicle control or 0.01 – 30 µg/kg TCDD every 4 days for 28 days using either (**A**) scBT or (**B**) WRS. Genes were ranked according to significance value and underwent gene set enrichment analysis using gene sets from BIOCARTA, KEGG, PANTHER, and WIKIPATHWAYS containing 15 – 250 genes. Enrichment was considered significant when adjusted p-value ≤ 0.05. Gene sets with ≥ 50% overlap in gene membership were considered connected to generate a network of closely related enriched terms. Individual clusters were manually annotated based on overall term names.

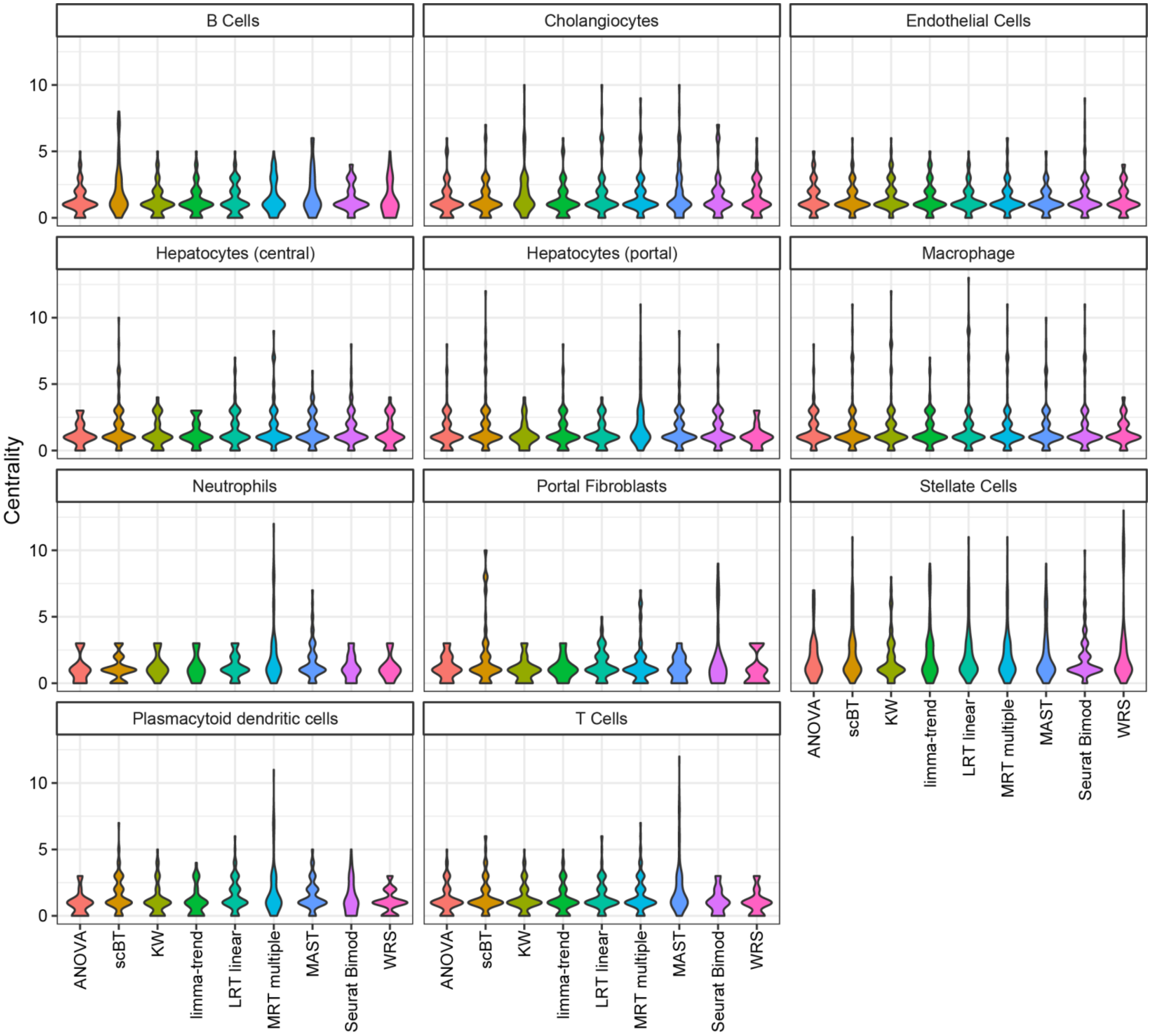

Supplementary Figure 28: Node-wise centrality of gene set enrichment analysis networks determined for each DGEA test method with hepatic single-nuclei RNA sequencing data from male mice gavaged with sesame oil vehicle control or 0.01 – 30 µg/kg TCDD every 4 days for 28 days. Genes were ranked according to significance value and underwent gene set enrichment analysis using gene sets from BIOCARTA, KEGG, PANTHER, and WIKIPATHWAYS containing 15 – 250 genes. Enrichment was considered significant when adjusted p-value ≤ 0.05. Gene sets with ≥ 50% overlap in gene membership were considered connected to generate a network of closely related enriched terms. Violin plots show the distribution of node-wise measurements.

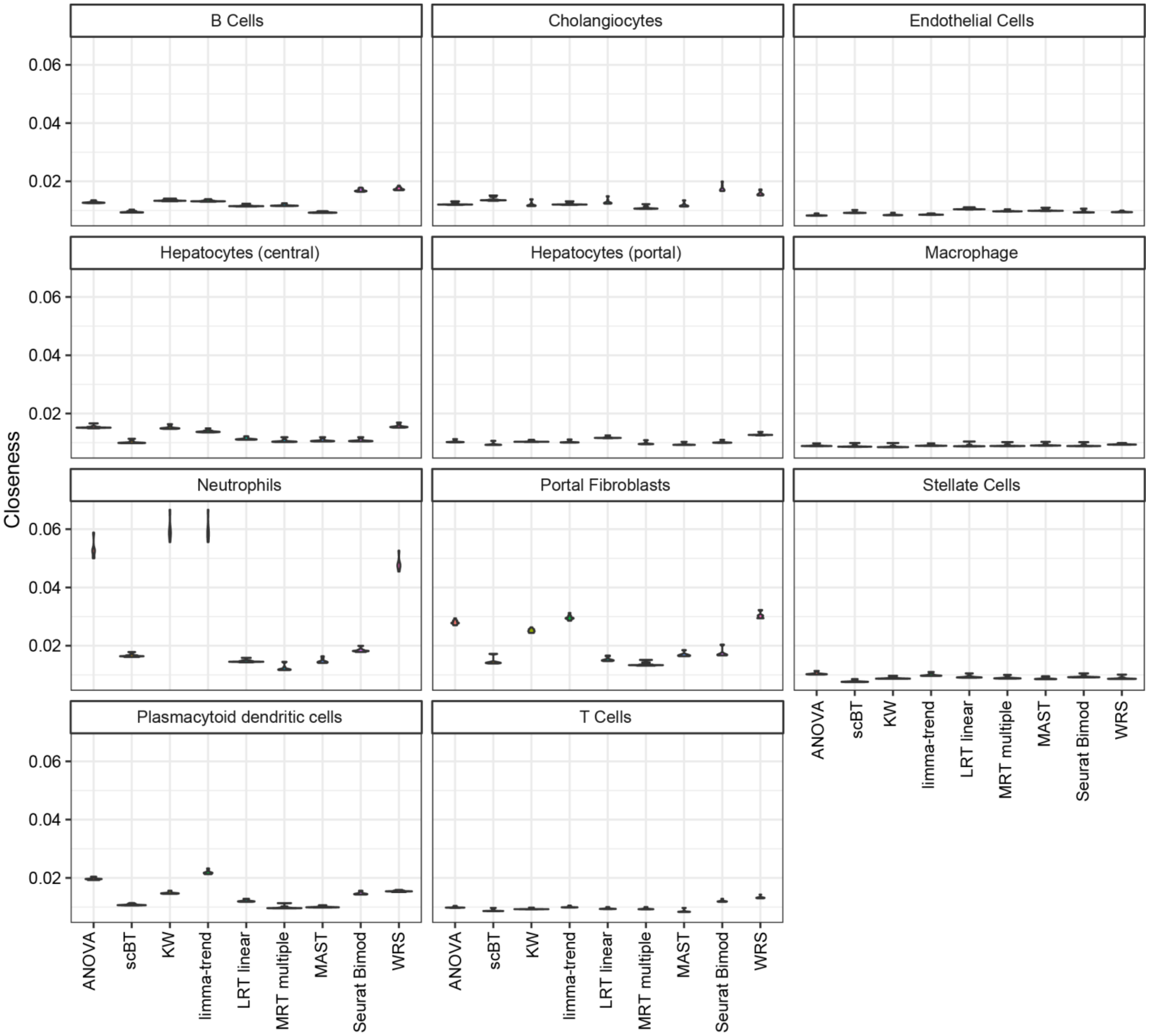

Supplementary Figure 29: Node-wise closeness of gene set enrichment analysis networks determined for each DGEA test method with hepatic single-nuclei RNA sequencing data from male mice gavaged with sesame oil vehicle control or 0.01 – 30 µg/kg TCDD every 4 days for 28 days. Genes were ranked according to significance value and underwent gene set enrichment analysis using gene sets from BIOCARTA, KEGG, PANTHER, and WIKIPATHWAYS containing 15 – 250 genes. Enrichment was considered significant when adjusted p-value ≤ 0.05. Gene sets with ≥ 50% overlap in gene membership were considered connected to generate a network of closely related enriched terms. Violin plots show the distribution of node-wise measurements.

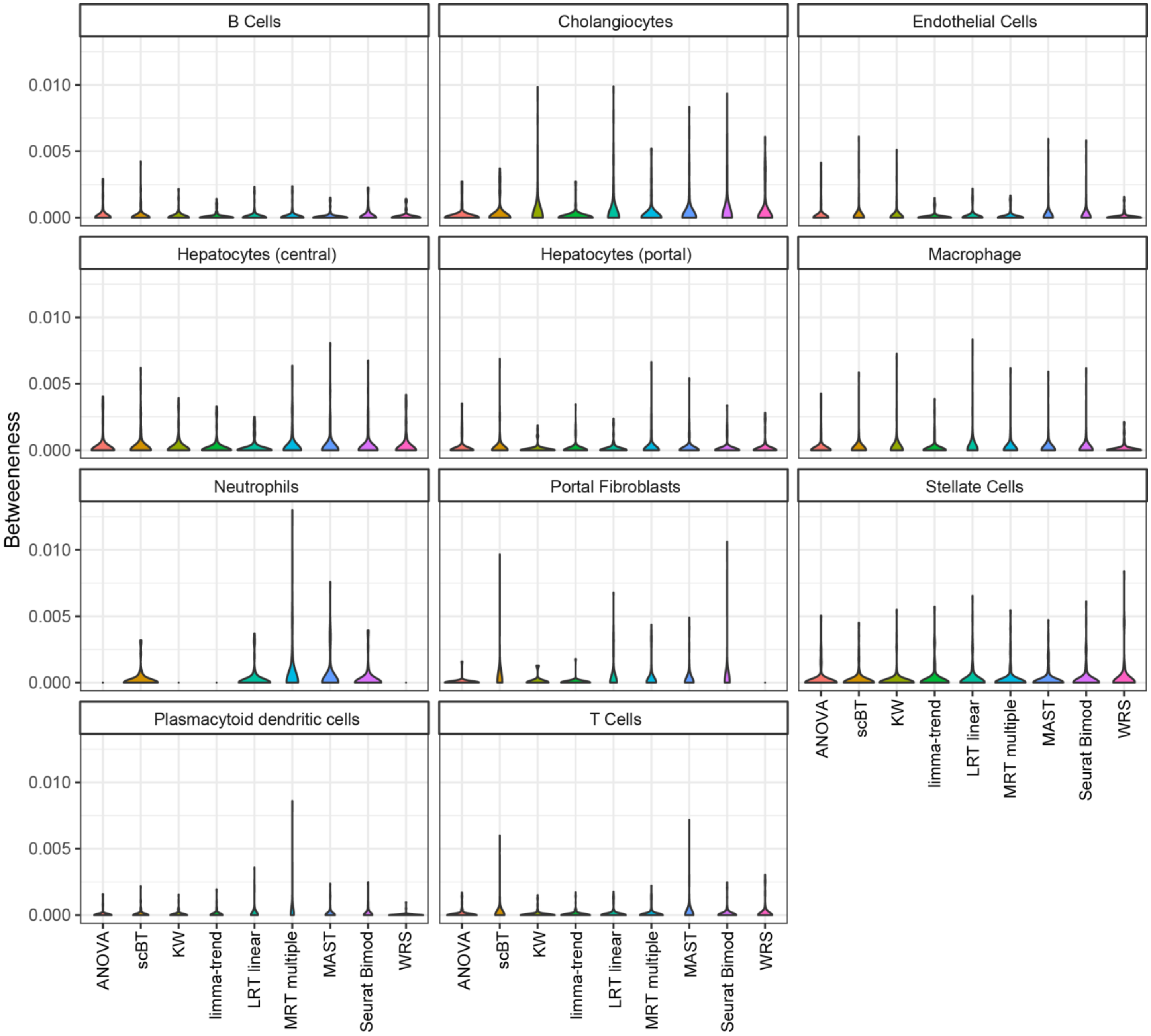

Supplementary Figure 30: Node-wise betweenness of gene set enrichment analysis networks determined for each DGEA test method with hepatic single-nuclei RNA sequencing data from male mice gavaged with sesame oil vehicle control or 0.01 – 30 µg/kg TCDD every 4 days for 28 days. Genes were ranked according to significance value and underwent gene set enrichment analysis using gene sets from BIOCARTA, KEGG, PANTHER, and WIKIPATHWAYS containing 15 – 250 genes. Enrichment was considered significant when adjusted p-value ≤ 0.05. Gene sets with ≥ 50% overlap in gene membership were considered connected to generate a network of closely related enriched terms. Violin plots show the distribution of node-wise measurements.
