# Supplementary material for: Benchmarking of a Bayesian single cell RNAseq differential gene expression test for dose–response study designs
Source: Nucleic Acids Res. 2022 Jan 21;50(8):e48. doi: 10.1093/nar/gkac019 (PMC9071439; doi:10.1093/nar/gkac019)
Supplement: gkac019_Supplemental_Files [file gkac019_supplemental_files.zip › scBT_NatMethods_SupplementalMethods.pdf]

Benchmarking of a Bayesian single cell RNAseq differential gene expression test for dose-response study designs- Supplementary Material

Contents

1 Derivation of the marginal likelihoods under the null and alternative hypothesis for scBA **1**

2 Derivation of the combined Likelihood Ratio Test Statistic (LRT-multiple) **3**

3 Derivation of the combined Likelihood Ratio Test Statistic for the linear model setup (LRT-linear) **4**

1 Derivation of the marginal likelihoods under the null and alternative hypothesis for scBA

Consider the following K-sample test,

$$H_0: \qquad \mu_{1,j} = \mu_{2,j} = \dots \mu_{K,j} = \mu_j \text{ and } \omega_{1,j} = \omega_{2,j} = \dots \omega_{K,j} = \omega_j. \tag{1}$$

versus the alternative

$$\begin{aligned} H_a: \qquad & \mu_{k,j} \text{ is different for at least one } k \text{ and} \\ & \omega_{k,j} \text{ is different for at least one } k, k = 1, \dots K. \end{aligned}$$

Given this model structure we sssume that a priori, given σ_j^2, $\mu_{k,j} \sim \text{Normal}(m_{k,0}, \tau_{k,\mu}\sigma_j^2)$, $\sigma_j^2 \sim IG(a_\sigma, b_\sigma)$, $\omega_{k,j} \sim \text{Beta}(a_{k,\omega}, b_{k,\omega})$, where IG is the inverse gamma distribution with shape a_σ and scale b_σ and $m_{k,0}$, $\tau_{k,\mu}, a_\sigma, b_\sigma, a_{k,\omega}, b_{k,\omega}$ are the hyperparameters. Now, let's assume that data are collected under K conditions, and denote the data by $D_{k,o} \equiv \{(Y_{k,i,j}, R_{k,i,j}), i = 1, \dots, n_k\}$. The underlying populations for the sample data $D_{k,o}$ for the k=1,2, . . . , K, are assumed to be identified by the parameters $(\mu_{k,j}, \sigma_j^2, \omega_{k,j})$. Under the null hypothesis $\mu_{1,j} = \mu_{2,j} = \dots \mu_{K,j} = \mu_j$ and $\omega_{1,j} = \omega_{2,j} = \dots \omega_{K,j} = \omega_j$. We sssume that a priori, given σ_j^2, $\mu_{k,j} \sim \text{Normal}(m_{k,0}, \tau_{k,\mu}\sigma_j^2)$, and

$\sigma_j^2 \sim IG(a_\sigma, b_\sigma)$, $\omega_{k,j} \sim \mathrm{Beta}(a_{k,\omega}, b_{k,\omega})$, where $m_{k,0}, \tau_{k,\mu}, a_\sigma, b_\sigma, a_{k,\omega}, b_{k,\omega}$ are the hyperparameters. Now we calculate the marginal likelihood under the null hypothesis and alternative hypothesis. Under the null hypothesis the marginal likelihood is

$$\begin{aligned}
\mathcal{L}_{H_0,j} &= \int\int\int \prod_{k=1}^{K} \left\{ \prod_{i=1}^{n_k} \left[\frac{1}{\sqrt{2\pi}\sigma_j} \exp\left\{ -\frac{(Y_{k,i,j}-\mu_j)^2}{2\sigma_j^2} \right\} \omega_j \right]^{R_{k,i,j}} (1-\omega_j)^{1-R_{k,i,j}} \right\} \\
&\quad \times \pi(\mu_j|\sigma_j^2)\pi(\sigma_j^2)\pi(\omega_j) d\mu_j d\sigma_j^2 d\omega_j \\
&= \frac{1}{(2\pi)^{(\sum_{k=1}^{K}\sum_{i=1}^{n_k} R_{k,i,j})/2}} \times \frac{1}{\sqrt{1+\tau_\mu \sum_{k=1}^{K}\sum_{i=1}^{n_k} R_{k,i,j}}} \\
&\quad \times \frac{1}{\Gamma(a_\sigma) b_\sigma^{a_\sigma}} \times \frac{\Gamma(a_\sigma + (\sum_{k=1}^{K}\sum_{i=1}^{n_k} R_{k,i,j})/2)}{(1/b_\sigma + \mathcal{A}_{tot}/2)^{a_\sigma + (\sum_{k=1}^{K}\sum_{i=1}^{n_k} R_{k,i,j})/2}} \\
&\quad \times \frac{\mathrm{Beta}(a_\omega + (\sum_{k=1}^{K}\sum_{i=1}^{n_k} R_{k,i,j}), b_\omega + \sum_{k=1}^{K} n_k - (\sum_{k=1}^{K}\sum_{i=1}^{n_k} R_{k,i,j}))}{\mathrm{Beta}(a_\omega, b_\omega)},
\end{aligned}$$

where

$$\mathcal{A}_{tot} = \left\{ \sum_{k=1}^{K}\sum_{i=1}^{n_k} R_{k,i,j} Y_{k,i,j}^2 + \frac{m_0^2}{\tau_\mu} \right\} - \left\{ \sum_{k=1}^{K}\sum_{i=1}^{n_k} R_{k,i,j} + \frac{1}{\tau_\mu} \right\}^{-1} \left\{ \sum_{k=1}^{K}\sum_{i=1}^{n_k} R_{k,i,j} Y_{k,i,j} + \frac{m_0}{\tau_\mu} \right\}^2.$$

Under the alternative hypothesis we compute the marginal likelihood without any restriction on the K means $\mu_{k,j}$ and the zero inflation parameter $\omega_{k,j}; k = 1, 2, \ldots K$. Particularly, we assume that $\mu_{k,j} \sim \mathrm{Normal}(m_{k,0}, \tau_{k,\mu}\sigma_j^2)$, and $\sigma_j^2 \sim IG(a_\sigma, b_\sigma)$, $\omega_{k,j} \sim \mathrm{Beta}(a_{k,\omega}, b_{k,\omega}); k = 1, 2, \ldots K$. Now,

$$\begin{aligned}
\mathcal{L}_{H_a,j} &= \int\cdots\int \left\{ \prod_{k=1}^{K}\prod_{i=1}^{n_k} \left[\frac{1}{\sqrt{2\pi}\sigma_j} \exp\left\{ -\frac{(Y_{k,i,j}-\mu_{k,j})^2}{2\sigma_j^2} \right\} \omega_{k,j} \right]^{R_{k,i,j}} (1-\omega_{k,j})^{1-R_{k,i,j}} \right\} \\
&\quad \times \prod_{k=1}^{K} \left\{ \pi(\mu_{k,j})\pi(\omega_{k,j}) \right\} \pi(\sigma_j^2) \prod_{k=1}^{K} \left\{ d\mu_{k,j} d\omega_{k,j} \right\} d\sigma_j^2 \\
&= \frac{1}{(2\pi)^{(\sum_{k=1}^{K}\sum_{i=1}^{n_k} R_{k,i,j})/2}} \times \frac{1}{\prod_{k=1}^{K} \sqrt{1+\tau_{k,\mu}\sum_{i=1}^{n_k} R_{k,i,j}}} \\
&\quad \times \frac{1}{\Gamma(a_\sigma) b_\sigma^{a_\sigma}} \times \frac{\Gamma(a_\sigma + \sum_{k=1}^{K}\sum_{i=1}^{n_k} R_{k,i,j}/2)}{(1/b_\sigma + \sum_{k=1}^{K} \mathcal{A}_k/2)^{a_\sigma + \sum_{k=1}^{K}\sum_{i=1}^{n_k} R_{k,i,j}/2}} \\
&\quad \times \prod_{k=1}^{K} \frac{\mathrm{Beta}(a_{k,\omega} + \sum_{i=1}^{n_k} R_{k,i,j}, b_{k,\omega} + n_k - \sum_{i=1}^{n_k} R_{k,i,j})}{\mathrm{Beta}(a_{k,\omega}, b_{k,\omega})},
\end{aligned}$$

where

$$\mathcal{A}_k = \left\{ \sum_{i=1}^{n_k} R_{k,i,j} Y_{k,i,j}^2 + \frac{m_{k,0}^2}{\tau_{k,\mu}} \right\} - \left\{ \sum_{i=1}^{n_k} R_{k,i,j} + \frac{1}{\tau_{k,\mu}} \right\}^{-1} \left\{ \sum_{i=1}^{n_k} R_{k,i,j} Y_{k,i,j} + \frac{m_{k,0}}{\tau_{t,\mu}} \right\}^2 \text{ for } k = 1, 2 \ldots K.$$

The ratio of the marginal likelihood from H_0 to H_a is

$$\frac{\mathcal{L}_{H_0,j}}{\mathcal{L}_{H_a,j}} = \frac{\prod_{k=1}^{K}\sqrt{1+\tau_{k,\mu}\sum_{i=1}^{n_k}R_{k,i,j}}}{\sqrt{1+\tau_{\mu}\sum_{k=1}^{K}\sum_{i=1}^{n_k}R_{k,i,j}}}$$
$$\times \frac{(1/b_\sigma + \sum_{k=1}^{K}\mathcal{A}_k/2)^{a_\sigma+\sum_{k=1}^{K}\sum_{i=1}^{n_k}R_{k,i,j}/2}}{(1/b_\sigma + \mathcal{A}_{tot}/2)^{a_\sigma+\sum_{k=1}^{K}\sum_{i=1}^{n_k}R_{k,i,j}/2}}$$
$$\times \frac{\mathrm{Beta}(a_\omega+\sum_{k=1}^{K}\sum_{i=1}^{n_k}R_{k,i,j}, b_\omega+\sum_{k=1}^{K}n_k-\sum_{k=1}^{K}\sum_{i=1}^{n_k}R_{k,i,j})}{\mathrm{Beta}(a_\omega, b_\omega)}$$
$$\times \prod_{k=1}^{K}\frac{\mathrm{Beta}(a_{k,\omega}, b_{k,\omega})}{\mathrm{Beta}(a_{k,\omega}+\sum_{i=1}^{n_k}R_{k,i,j}, b_{k,\omega}+n_k-\sum_{i=1}^{n_k}R_{k,i,j})}.$$

The Bayes factor can be thus be defined as

$$BF_{01,j} = \frac{\mathcal{L}_{H_0,j}}{\mathcal{L}_{H_a,j}} \times \frac{\pi(H_a)}{\pi(H_0)},$$

where $\pi(H_a)$ and $\pi(H_0)$ are the prior probabilities for the alternative and null model, respectively. To control for multiplicity we adopt the FDR correction approach discussed in[1]. The rejection threshold is estimated in terms of the posterior probabilities of the null hypothesis, $p(H_{0,j}|D_j)$. For a target FDR α, the procedure rejects all hypotheses with $p(H_{0,j}|D_j) < \zeta$, where $p(H_{0,j}|D_j) = (1+1/BF_{01,j})^{-1}$ and ζ is the largest value such that $C(\zeta)/J(\zeta) \leq \alpha$ where, $J(\zeta) = \{j : p(H_{0,j}|D_j) \leq \zeta\}$ and $C(\zeta) = \sum_{j\in J(\zeta)} p(H_{o,j}|D_j)$.

2 Derivation of the combined Likelihood Ratio Test Statistic (LRT-multiple)

In this section, we extend the two-sample test proposed by[2] to a test for k-samples. Consider the composite K-sample test

$$H_0 : \omega_1 = \omega_2 = \cdots = \omega_K = \omega \quad \text{and} \quad \mu_1 = \mu_2 = \cdots = \mu_K = \mu$$

versus the alternative

$$H_a : \omega_k \text{ is different for at least one } k \quad \text{and} \quad \mu_k \text{ is different for at least one } k, \quad k \in 1, \ldots, K.$$

Similar to ANOVA, we assume homogeneity for variance parameter σ_j^2. Now, fixing the gene index j, the likelihood ratio test can be defined as;

$$\Lambda(Y, R) = \frac{\sup_{\theta\in H_0} L(\theta|Y, R)}{\sup_{\theta\in H_a} L(\theta|Y, R)}$$

where the likelihood can be written as;

$$L(\theta|Y,R) = \prod_k \omega_k^{e_k}(1-\omega_k)^{n_k-e_k}\prod_{i\in C_k} f(Y_{i,k}|\mu_k,\sigma^2)$$

Y and R represent the gene observation vector and the gene indicator vector across K dose groups and $\theta = \{\mu_k, \sigma^2, \pi_k, k = 1, \ldots, K\}$ is the vector of unknown parameters. We define C_k to be the set of cells expressing the gene in group k $(i.e. C_k = \{i : R_{ik} = 1\})$ and $e_k = \sum_i R_{ik}$ is the cardinality of set C_k. Here, f denotes the density function of the normal distribution with parameters μ_k and σ^2. Therefore, it follows that the likelihood ratio test can be written as

$$\begin{aligned}
\Lambda(Y,R) &= \frac{\sup_{\theta\in H_0} L(\theta|Y,R)}{\sup_{\theta\in H_a} L(\theta|Y,R)}\\
&= \frac{\sup_{\{\omega,\mu,\sigma^2\}} \omega^{(\sum_k e_k)}(1-\omega)^{(\sum_k n_k - \sum_k e_k)}\prod_k\prod_{i\in C_k} N(Y_{i,k}|\mu,\sigma^2)}{\sup_{\{\omega_k,\mu_k,\sigma^2;k=1,\ldots,K\}}\prod_k \omega_k^{e_k}(1-\omega_k)^{(n_k-e_k)}\prod_k\prod_{i\in C_k} N(Y_{i,k}|\mu_k,\sigma^2)}\\
&= \frac{\sup_{\{\omega\}} \omega^{(\sum_k e_k)}(1-\omega)^{(\sum_k n_k - \sum_k e_k)}}{\sup_{\{\omega_k,k=1,\ldots,K\}}\prod_k \omega_k^{e_k}(1-\omega_k)^{(n_k-e_k)}} \times \frac{\sup_{\{\mu,\sigma^2\}}\prod_k\prod_{i\in C_k} N(Y_{i,k}|\mu,\sigma^2)}{\sup_{\{\mu_k,\sigma^2;k=1,\ldots,K\}}\prod_k\prod_{i\in C_k} N(Y_{i,k}|\mu_k,\sigma^2)}\\
&= \prod_k \left\{\frac{\frac{\sum_k e_k}{\sum_k n_k}}{\frac{e_k}{n_k}}\right\}^{e_k} \times \left\{\frac{1-\frac{\sum_k e_k}{\sum_k n_k}}{1-\frac{e_k}{n_k}}\right\}^{n_k-e_k} \times \left\{1 + \frac{\sum_k e_k(\bar{Y}_k^+ - \bar{\bar{Y}}^+)^2}{\sum_k\sum_{i=1}^{e_k}(Y_{ik}^+ - \bar{Y}_k^+)^2}\right\}^{-\frac{\sum_k e_k}{2}}\\
&= \Lambda_b(R)\times\Lambda_n(Y^+),
\end{aligned}$$

where $N(\cdot|\mu,\sigma^2)$ denotes the normal density with mean and variance μ and σ^2, Λ_b is a binomial LRT, Λ_n is a normal LRT ,Y^+ is the set of positive Y values, $\overline{Y_k^+} = (1/e_k)\sum_{i=1}^{e_k} Y_{ik}^+$ and $\overline{\overline{Y^+}} = (1/\sum_k e_k)\sum_k\sum_{i=1}^{e_k} Y_{ik}^+$. Thus our combined LRT can be computed as the product of a binomial and a normal LRT statistic, both of which can easily be derived using classical statistical theory.

3 Derivation of the combined Likelihood Ratio Test Statistic for the linear model setup (LRT-linear)

In this section we extend the combined Likelihood Ratio Test Statistic (LRT-multiple) to a linear model setup. Treating dose (d) as a continuous covariate we write $\mu_{ij} = m_{0j} + d_i m_{1j}$ and $\text{logit}(\omega_{ij}) = \psi_{0j} + d_i\psi_{1j}$. Under the null hypothesis the model can be reformulated as $H_0 : \mu_{ij} = m_{0j}$ and $\text{logit}(\omega_{ij}) = \psi_{0j}$. Therefore the likelihood function for gene j under the full model can be written as:

$$L(\theta_j|Y_j,R_j) = \prod_{i=1}^{n}\frac{\{\exp(\psi_{0j}+d_i\psi_{1j})\}^{R_{ij}}}{1+\exp(\psi_{0j}+d_i\psi_{1j})}\prod_{i=1}^{n_e} N(Y_{ij}^+|\mu_{ij} = m_{0j} + d_i m_{1j}, \sigma_j^2),$$

where $R_j = I(Y_j \neq 0)$ denotes the gene expression indicator vector of size $n = \sum_{k=1}^{K} n_k$, Y_j^+ denotes the positively expressed gene observation vector of size $n_e = \sum_k \sum_i R_{ijk}$ and $\theta_j = \{m_{0j}, m_{1j}, \sigma_j^2, \psi_{0j}, \psi_{1j}\}$ Using the likelihood function described above, the likelihood ratio test can be derived following the same approach detailed in Section 2. Since R_j and Y_j are conditionally independent for each gene j, the individuals LRT statistics derived from the logistic and linear regression parts can be summed to obtain an asymptotically χ^2 distribution with the degrees of freedom of the component tests added.

References

[1] M. A. Newton, A. Noueiry, D. Sarkar, and P. Ahlquist. Detecting differential gene expression with a semiparametric hierarchical mixture method. *Biostatistics*, 5(2):155–76, 2004.

[2] Andrew McDavid, Greg Finak, Pratip K Chattopadyay, Maria Dominguez, Laurie Lamoreaux, Steven S Ma, Mario Roederer, and Raphael Gottardo. Data exploration, quality control and testing in single-cell qpcr-based gene expression experiments. *Bioinformatics*, 29(4):461–467, 2013.
